# Type I partition-related proteins enhance conjugative transfer through transcriptional activation and *oriT* region binding

**DOI:** 10.1128/mbio.01600-25

**Published:** 2025-07-31

**Authors:** Kouhei Kishida, Koji Kudo, Ren Kumagai, Sakura Ijima, Wenhao Deng, Leonardo Stari, Natsumi Ogawa-Kishida, Yoshiyuki Ohtsubo, Yuji Nagata, Masataka Tsuda

**Affiliations:** 1 Department of Molecular and Chemical Life Sciences, Graduate School of Life Sciences, Tohoku University 13101, Sendai, Japan; Harvard Chan School of Public Health, Boston, Massachusetts, USA

**Keywords:** plasmid, conjugative transfer, partition system, DNA binding protein, *Pseudomonas*

## Abstract

Plasmid partitioning and bacterial conjugation are critical processes ensuring plasmid maintenance and dissemination, respectively, within bacterial populations. Although traditionally regarded as distinct phenomena, these two processes are increasingly recognized as interconnected. While partitioning ensures plasmid inheritance during cell division, its potential influence on conjugative transfer remains poorly understood. A major impediment to understanding their interplay is that partition systems are often essential for plasmid stability, making it difficult to distinguish their direct effects on conjugation. In this study, we addressed this challenge using a mini-conjugative plasmid derived from the *Pseudomonas putida* NAH7 plasmid. This engineered plasmid, containing all conjugation-related genes, was cloned into an *Escherichia coli*-compatible vector. Additionally, the *par* genes from NAH7 were expressed from a separate plasmid to investigate their roles in conjugative transfer. Our results revealed that the *par* gene cluster plays a significant role in enhancing the conjugative transfer of the mini-conjugative plasmid. Specifically, ParB, a centromere-binding protein, functions as a transcriptional activator of conjugation-related genes by binding the *parS_NAH_* site. In contrast, ParR, a KorA homolog, was not found to enhance transcription directly but binds extensively to the *oriT* region. This binding probably facilitates the recruitment or stabilization of the relaxosome, thereby enhancing conjugation efficiency. Together, these findings unveil a previously unappreciated role for partition proteins in stimulating bacterial conjugation, providing new insights into how plasmids coordinate vertical and horizontal dissemination, highlighting that these processes can occur simultaneously within bacterial communities.

**IMPORTANCE:**

Plasmid partition systems are classified into three types. While some systems have been reported to influence conjugative transfer, this study uncovers a novel mechanism utilized by a Type I system to enhance DNA transfer. Strikingly, repeat sequences perfectly matching the *parS_NAH_* site—bound by ParB to activate downstream conjugative transfer genes—were identified on both plasmids and chromosomes across diverse proteobacterial taxa. Furthermore, many of these repeat sequences were localized near genes involved in conjugative transfer and partitioning, suggesting the presence of a conserved regulatory mechanism mediated by these repeats. This study provides important insights into how plasmid partition systems coordinate both vertical and horizontal dissemination. Such knowledge is essential for understanding and mitigating the spread of antibiotic resistance and other plasmid-encoded traits, and it offers a foundation for developing strategies to manage plasmid-associated genetic exchange.

## INTRODUCTION

The propagation of plasmids through conjugative transfer is a biologically significant phenomenon. It contributes to the emergence of antibiotic-resistant and pollutant-degrading bacteria (1), as well as the dissemination of virulence-associated plasmids, which are essential for host invasion and pathogenicity (2). These diverse functional roles highlight the need to understand the molecular mechanisms that activate conjugative transfer. In gram-negative bacteria, conjugation involves two main steps. First, the DNA transfer and replication (Dtr) system, in which relaxases serve as key enzymes, binds to and processes the cognate origin of transfer (*oriT*) DNA region, generating transfer-DNA (T-DNA) (1). The *oriT* DNA region is not only bound by relaxase but also by accessory proteins and transcriptional regulators that act on promoters located near the *oriT* region (3–5). Second, the conjugative proteins assemble into the type IV secretion system (T4SS) channel through which T-DNA is transferred to recipient cells (6, 7).

Low-copy-number plasmids are stably maintained by partition mechanisms that are responsible for their proper positioning within the cell during cell division (8, 9). Plasmid partition systems (Par systems) generally comprise two essential proteins and partition sites (*par* sites) on plasmid DNA (8). One of these proteins, the centromere-binding protein (CBP), is a site-specific DNA-binding protein that recognizes the *par* sites. The other protein is an NTPase (ATPase or GTPase), which supplies the necessary energy to facilitate the movement of plasmid DNA within the cell. Par systems are classified into three types based on their NTPase properties: Type I (Walker-type ATPases), Type II (actin-like ATPases), and Type III (tubulin-like GTPases) (10, 11).

Par-related proteins (Par proteins) play roles in regulating conjugative transfer by repressing the transcription of conjugation-related genes and interacting with conjugation-associated proteins (12–15). Although Par proteins have been shown to enhance conjugative DNA transfer, the overall understanding of their roles in this process remains limited. One reason for this limitation is that disruption or overexpression of *par* genes inherently affects plasmid stability, making it difficult to isolate their direct effects on conjugation efficiency.

IncP-9 plasmid, which is primarily maintained in *Pseudomonas* species, often carries accessory genes, including antibiotic resistance genes and genes involved in the degradation of polycyclic aromatic hydrocarbons (16, 17). The IncP-9 naphthalene-catabolic plasmid NAH7 has been extensively studied for its conjugative transfer capabilities (18–23), and its naphthalene degradation genes are carried on the transposable element Tn*4655*. It was originally isolated from *Pseudomonas putida* G7 (24), where it enables the host bacterium to utilize naphthalene as a source of carbon and energy. In practice, NAH7 can replicate and be maintained in both *Pseudomonas* and *Escherichia coli*, and it is capable of conjugative transfer not only to gamma-proteobacteria but also to alpha- and beta-proteobacteria, demonstrating a broad host range and further emphasizing its potential impact on microbial ecology and bioremediation (18). We have identified fundamental properties of NAH7 related to its conjugation system, including the *oriT*, its *nic* site, and the *relaxase* gene (18). pKKO3-2, a plasmid we constructed by cloning all the conjugation-related genes from NAH7 into the non-mobilizable shuttle vector pNIT6012 (25), has been demonstrated to be capable of conjugative transfer (19). Since pKKO3-2 does not contain the *par* genes from NAH7, it is suitable for investigating whether the *par* genes enhance conjugative transfer.

In this study, we demonstrated that the *par* gene cluster from the NAH7 Type I partition system, which includes ParB as a CBP and ParR, the KorA homolog, contributes not only to plasmid stability but also to the activation of conjugative transfer. We found that ParB functions as a transcriptional activator for conjugation-related genes, likely through its binding to *parS_NAH_* sites. Although ParR did not exhibit transcriptional activation, it was observed to bind a broad region of the *oriT* region, suggesting that this association contributes to the enhancement of conjugative transfer. Together, our findings highlight a previously unappreciated role for Type I partition systems in stimulating conjugative transfer.

## RESULTS

### Identification of plasmid partition-related genes and *par* sites on NAH7

**Fig 1:**
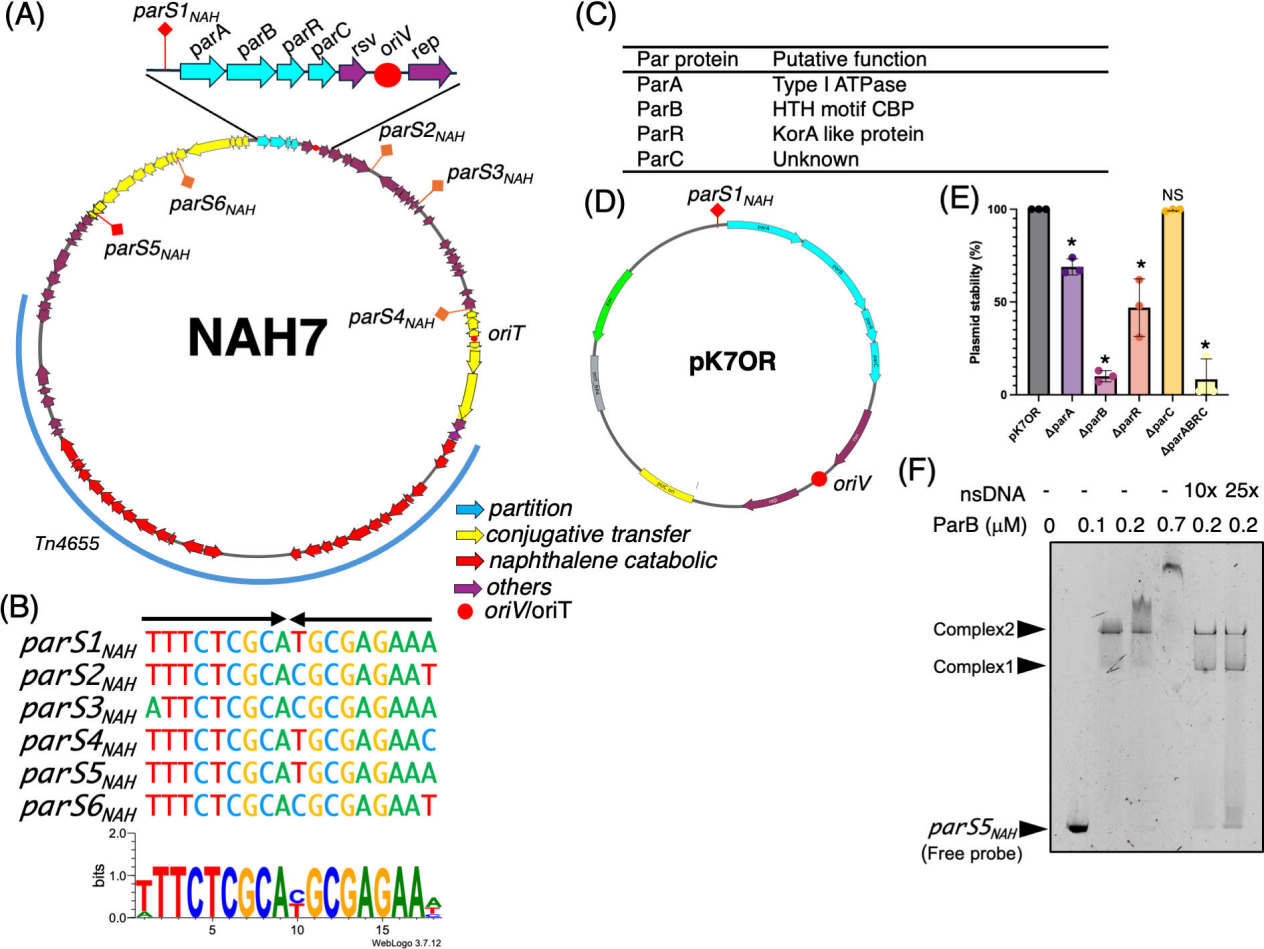
Overview of NAH7 plasmid partition system and characterization of *parS* sites and Par proteins. (**A**) Circular map of NAH7. Partitioning genes (*parA*, *parB*, *parR*, and *parC*) are indicated in cyan, conjugative transfer-related genes are indicated in yellow, naphthalene catabolic genes are shown in red, and other genes are shown in purple. The origin of replication (*oriV*) and origin of transfer (*oriT*) are marked with red circles. Six inverted repeat sequences, designated *parS1_NAH_* through *parS6_NAH_*, are distributed across the plasmid (marked in orange or red). (**B**) Alignment of the identified *parS_NAH_* sites. *parS_NAH_* sequences are shown with similarities highlighted, indicating conserved nucleotide patterns, *parS1_NAH_* and *parS5_NAH_* are perfect inverted repeat sequences, while the other *parS_NAH_* sites (*parS2_NAH_* through *parS4_NAH_*, *parS6_NAH_*) contain up to two mismatches. The sequence logo was generated using *parS1_NAH_* to *parS6_NAH_* with WebLogo (28). (**C**) Summary table listing the putative functions of the NAH7 partition proteins, based on predictions from InterPro analysis (Fig. S1). (**D**) Plasmid map of pK7OR, derived from pK18mob, highlighting key genetic elements. The plasmid features the *parS1_NAH_* site located upstream of the *parA*, *parB*, *parR*, and *parC* genes (cyan arrows). The *oriV* (red circle), *resolvase,* and *rep* genes (purple arrows) together support plasmid replication, ensuring its maintenance in host cells. The Km gene (green arrow) provides kanamycin resistance for selection. (**E**) Plasmid stability assay for pK7OR, with deletions of individual *par* genes (Δ*parA*, Δ*parB*, Δ*parR*, and Δ*parC*) and all four *par* genes, conducted in *P. putida* KT2440. Stability was measured as the percentage of plasmid-harboring (Km^R^) colonies out of 100 colonies after 24 hours of growth in non-selective conditions (approximately 10 generations). The assay was performed in three independent biological replicates. The average values were plotted with individual data points, and standard deviations are represented as error bars. Statistical analyses were performed by ordinary one-way ANOVA with Dunnett’s multiple comparisons test, comparing each deletion mutant with pK7OR (*P < 0.005). “NS” denotes no significant difference. (**F**) Electrophoretic mobility shift assay showing the binding of ParB-His protein to the *parS5_NAH_* DNA fragment. Each lane contains an 82 nM FAM-labeled *parS5_NAH_* DNA fragment. The final concentrations of ParB-His are shown in the figure. Non-specific competitor DNA (salmon sperm DNA [nsDNA]) was added at concentrations 10-fold and 25-fold higher than the FAM-labeled *parS5* DNA fragment. Samples were electrophoresed on a 15% polyacrylamide gel at 200 V for 90 minutes using Tris-glycine buffer. Complex 1 and Complex 2 represent the major shifted bands, while *parS5_NAH_* represents the free probe.

To gain foundational insights into the Par system of NAH7, we conducted a bioinformatics analysis of its partition-related genes and associated *par* sites (Fig. 1A through C). Annotation of the NAH7 plasmid (accession number NC_007926.1) (26, 27) revealed a gene cluster comprising *parA*, *parB*, *parR*, and *parC* (Fig. 1A). ParA was predicted to be an ATPase containing a Walker A motif, characterized by the consensus sequence KGGxxK[T/S] (11) (Fig. S1), suggesting NAH7 utilizes a Type I partitioning system, and ParB was predicted as a CBP (Fig. S1). In Type I partitioning systems, the *par* sites recognized by CBPs typically contain inverted repeat (IR) sequences (11). Guided by this information, we searched for IR sequences in regions surrounding the *par* genes on NAH7 to locate potential *par* sites. This analysis identified an 18-bp IR sequence, 5′-TTTCTCGCATGCGAGAAA-3′ (designated *parS1_NAH_*), located at positions 82,127–82,143 (Fig. 1A and B). Additionally, we discovered five other similar or identical IR sequences (*parS2_NAH_* to *parS6_NAH_*), each differing by up to two nucleotides from *parS1_NAH_*, distributed across NAH7 and located in intergenic regions. Notably, these IR sequences were located in the IncP-9 backbone; none of them were found within the Tn*4655* transposable element, suggesting that these IR sequences are essential components of the plasmid backbone. The *parS5_NAH_* sequence, positioned upstream of the *mpfK* gene—an essential gene for conjugative transfer in IncP-9 plasmids—was identical to *parS1_NAH_*, while the other IRs exhibited partial conservation (Fig. 1B). Further analysis predicted that ParR contains a KorA domain (Fig. S1), known to be involved in transcriptional regulation. Although the specific function of ParC remains unknown, it is conserved among IncP-9 plasmids (Table S2).

To investigate the role of these *par* genes in maintaining the stability of NAH7, we performed plasmid stability assays using mini-NAH7 variants lacking each *par* gene in *P. putida* KT2440 (Fig. 1D). The mini-NAH7 plasmid (pK7OR) was engineered by cloning the region spanning from *parS1_NAH_* to the *rep* gene into an *E. coli* plasmid vector containing a kanamycin resistance marker (Fig. 1D). The mini-NAH7 plasmid exhibited stable inheritance, with 100% of the progeny retaining the plasmid over approximately 10 generations. In contrast, the plasmid lacking all four genes (*parA*, *parB*, *parR*, and *parC*) showed the greatest reduction in stability. Deletions of *parA*, *parB*, and *parR* led to significantly reduced plasmid stability, whereas deletion of *parC* did not markedly affect stability. These results indicate that *parA*, *parB*, and *parR* are essential for plasmid stability, while *parC* is dispensable under the conditions tested. To minimize potential polar effects associated with full gene deletions, additional constructs were generated in which internal deletions (ParAΔ15-214, ParBΔ35-232, and ParRΔ35-79) or frameshift mutations were introduced into each gene within the pK7OR backbone (Fig. S1 and S2). All of these modified plasmids exhibited reduced stability compared to wild-type pK7OR, supporting the importance of *parA*, *parB*, and *parR* in plasmid maintenance. Moreover, complementation experiments showed that plasmid stability was restored by expressing *parB* or *parR* in *trans*, while complementation of *parA* was not successful. Nevertheless, the destabilizing effects of internal deletion and frameshift mutations in *parA*, along with bioinformatic evidence indicating its identity as a partitioning ATPase, support its essential role in plasmid stability.

To confirm that ParB functions as a CBP and that the *parS_NAH_* sequence acts as a *par* site on NAH7, we performed an electrophoretic mobility shift assay (EMSA) with a *parS_NAH_* DNA probe. Increasing concentrations of ParB resulted in the formation of two distinct DNA-protein complexes, designated Complex 1 and Complex 2. These complexes were also observed in the presence of an excess amount of non-specific DNA (nsDNA), such as salmon sperm DNA, indicating that ParB binds to *parS_NAH_* with high specificity. The presence of these complexes suggests that ParB binds to *parS_NAH_* in a multimeric form. These results support the conclusion that ParB is a CBP and that *parS_NAH_* functions as a *par* site essential for the partitioning system of NAH7.

### Enhanced conjugative transfer of pKKO3-2 by *par* genes

**Fig 2:**
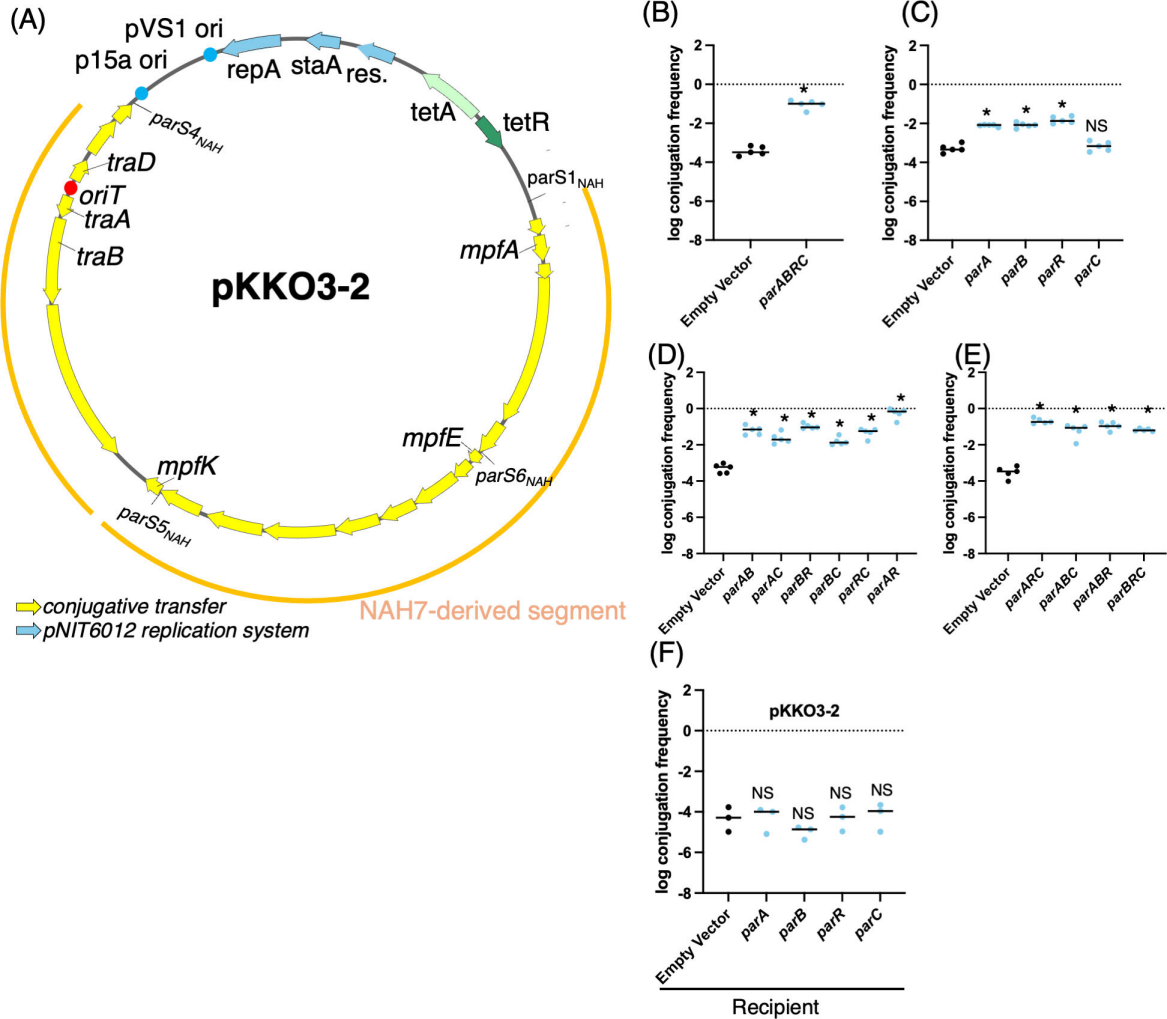
Effects of *par* genes on conjugative transfer of pKKO3-2. (**A**) Plasmid map of pKKO3-2. The yellow segments represent genes involved in conjugative transfer derived from NAH7, and the blue arrows represent the pNIT6012 replication system. The locations of four *parS_NAH_* sites are shown. (B–E) Conjugative transfer frequency of pKKO3-2 with the expression of different *par* genes from a pUC18 vector in donor cells. Panel (**B**) shows the effect of expressing all four *par* genes (*parA*, *parB*, *parR*, and *parC*) together, while panels (**C–E**) represent the effects of expressing different single *par* genes, as well as various combinations of two or three par genes. (**F**) Conjugative transfer frequency of pKKO3-2 with the expression of individual *par* genes from a pUC18 vector in recipient cells. Overnight-grown donor and recipient cells were mixed 1:1 and subjected to solid mating on LB agar containing IPTG at 30°C for 6 hours, after which CFUs on selective plates were counted. Conjugation frequency is shown on a log scale as the number of transconjugants per donor cell (Tc/D). Each experiment was performed at least five times independently. Error bars represent the standard deviation from biological replicates, and individual data points are shown. Statistical analysis for panel (**B**) was performed using an unpaired two-tailed *t*-test, while panels (**C–F**) were analyzed by ordinary one-way ANOVA followed by Dunnett’s multiple comparisons test relative to the strain harboring an empty vector. Asterisks (*P < 0.0001) indicate significant differences compared to Ev. “NS” denotes no significant difference.

We found that the identified *parS_NAH_* sequences were not only located around the *par* genes but were also positioned close to genes involved in conjugative transfer (Fig. 1A). This distribution suggests that there is a potential crosstalk between partitioning and conjugative transfer mechanisms in NAH7. Attempts to replace the *par* gene cluster in the NAH7 plasmid with a kanamycin resistance cassette yielded Km-resistant colonies. However, even after multiple rounds of single-colony isolation, every colony still harbored both wild-type NAH7 and the *par-*deleted plasmid (Fig. S3). This suggests that expression of the *par* genes may affect the overall transcriptional profile in a way that is essential for host cell viability, making it difficult for cells carrying only the par-deleted plasmid to survive. Therefore, we employed a plasmid pKKO3-2 to investigate how *par* genes influence conjugative transfer (Fig. 2). pKKO3-2 carries all of the conjugation-related genes from NAH7 but lacks these replication and partitioning genes, making it an ideal model for examining the direct effects of *par* genes on conjugative transfer. To assess the impact of *parABRC* on pKKO3-2 transfer between *E. coli* strains, we induced their expression from a pUC18 vector using isopropyl β-D-1-thiogalactopyranoside (IPTG). Under these conditions, the pKKO3-2 transfer frequency increased by more than 200-fold (Fig. 2B) without affecting donor cell growth. To identify which specific *par* genes were responsible for this enhancement (Fig. 2C), we expressed each gene individually. Expression of *parA*, *parB*, or *parR* significantly elevated transfer frequency, whereas *parC* did not. We further examined various two- and three-gene combinations (Fig. 2D and E), all of which produced higher transfer frequencies than any single gene alone. Notably, the *parA* and *parR* pair yielded the greatest increase among the two-gene sets, and among the three-gene combinations, *parARC* resulted in the highest transfer frequency. In contrast, expression of individual *par* genes in the recipient cells did not affect the transfer frequency (Fig. 2F). These findings indicate that *parA*, *parB*, and *parR* individually promote conjugative transfer, and that their combined expression can further intensify this effect in donor cells.

### Transcriptional analysis of conjugation-related genes in pKKO3-2 expressing individual *par* genes 

**Fig 3:**
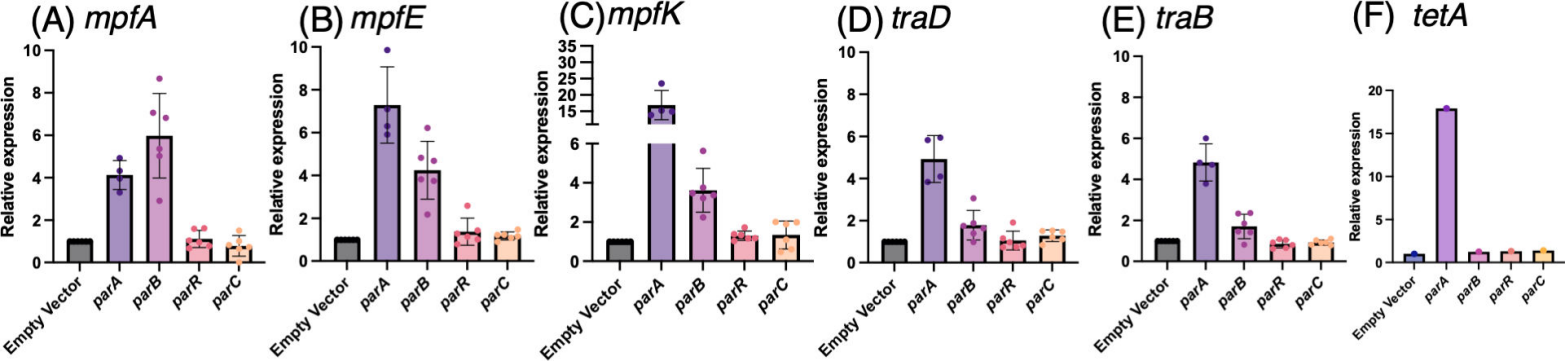
Relative expression levels of conjugative transfer genes in *E. coli* strains harboring the pKKO3-2 plasmid with individual *par* gene expression. (A–F) Gene expression levels were quantified using qRT-PCR. The graphs show the relative expression of (**A**) *mpfA*, (**B**) *mpfE*, (**C**) *mpfK*, (**D**) *traD*, (**E**) *traB,* and (**F**) *tetA*. The relative expression values are normalized to the empty vector strain, which is set as 1. The data represent the mean, with each dot corresponding to an independent biological replicate, and individual data points are shown. Error bars indicate standard deviation. For RNA extraction, cultures expressing each par gene were spotted onto IPTG-containing Lysogeny Broth agar plates and incubated before harvesting. RNA samples were treated with DNase I, and reverse transcription was performed using random primers. qRT-PCR was carried out using standard cycling conditions. Specific primer pairs were used to amplify each target gene, and expression levels were normalized to gyrA.

**Fig 4:**
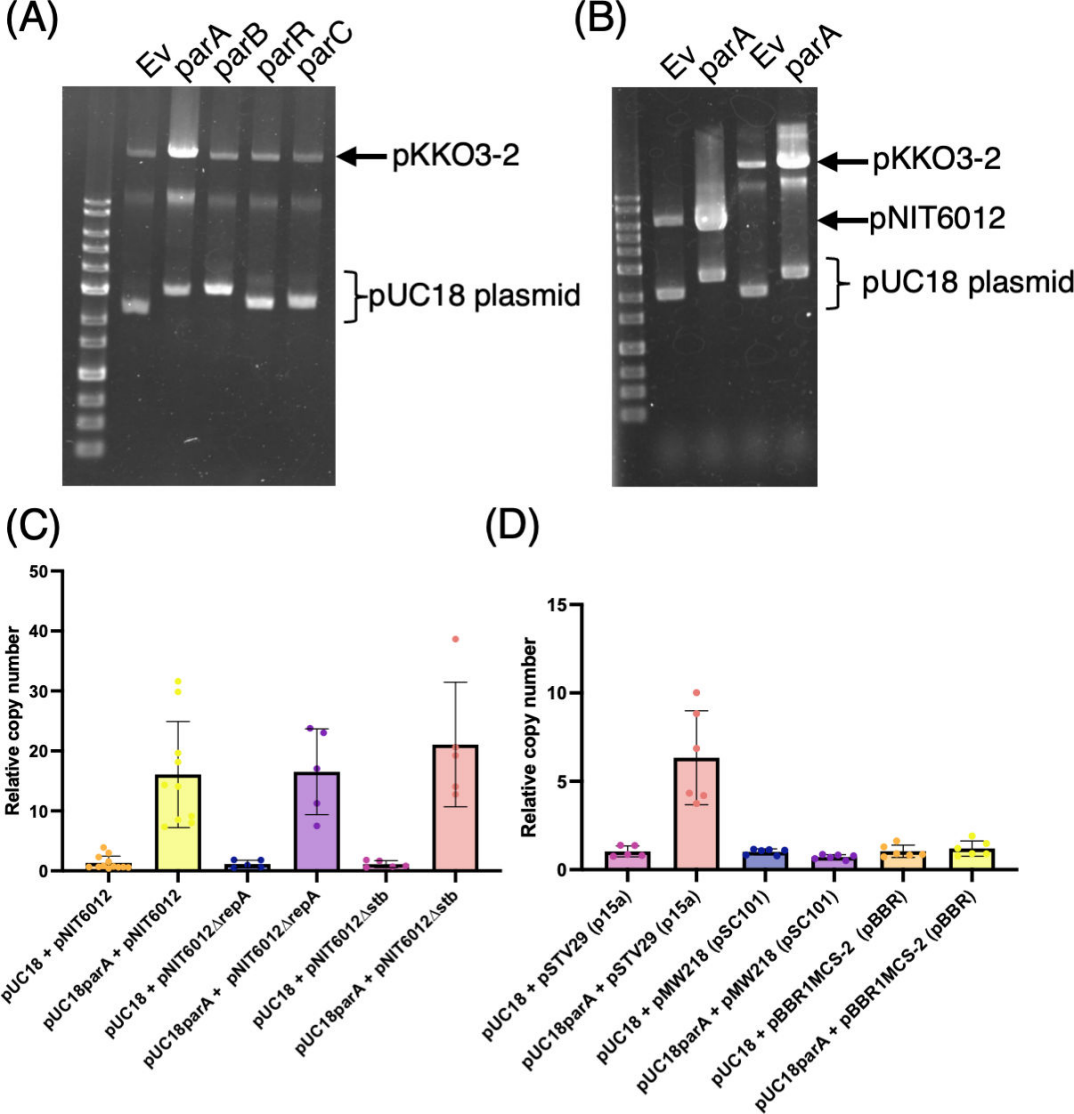
ParA specifically increases the copy number of p15A-based plasmids in *E. coli.* (**A and B**) Agarose gel electrophoresis of plasmid preparations from *E. coli* strains. (**A**) shows plasmid profiles for *E. coli* carrying pKKO3-2 and pUC18 derivatives expressing individual *par* genes (*parA*, *parB*, *parR*, or *parC*), as indicated above each lane. (**B**) shows plasmid profiles for *E. coli* harboring pNIT6012- and pUC18-based expression constructs. Positions of pKKO3-2, pNIT6012, and pUC18 plasmids are indicated. (**C and D**) Quantification of plasmid copy number by quantitative PCR. Relative plasmid copy number was calculated using *gyrA* as the internal chromosomal control and specific regions on each plasmid. Bars represent the average values from independent replicates, and error bars indicate standard deviations. The number of biological replicates is shown as individual dots. Detailed methods are described in Materials and Methods. Briefly, cells were grown on Lysogeny Broth agar plates with IPTG induction, and DNA was extracted by boiling the harvested cells.

Because several Par proteins are predicted to bind DNA, we hypothesized that they activate transcription of conjugation-related genes. To test this hypothesis, we used quantitative reverse transcription PCR (qRT-PCR) with *gyrA* as an internal standard to measure the transcription levels of several conjugation-related genes in *E. coli* strains harboring pKKO3-2 and expressing a single *par* gene from a pUC18-based vector. Overnight cultures were spotted onto IPTG-containing agar plates and incubated at 30°C for 3 hours prior to RNA extraction. We quantified the relative expression of *mpfA*, *mpfE*, *mpfK*, *traD*, and *traB* (Fig. 3). Our results showed that T4SS-related genes (*mpfA*, *mpfE*, and *mpfK*) were upregulated in strains expressing *parA* or *parB* (Fig. 3A through C). In contrast, neither *parR* nor *parC* affected the transcription of any T4SS-related genes. We next examined *traD* and *traB*, which have promoters located on *oriT*. Here, an increase in transcription was observed only with *parA*, while the other *par* genes had no noticeable effect (Fig. 3D and E). *parA* increased the transcription of all conjugation-related genes tested. Interestingly, *parA* also elevated *tetA* transcription in the pNIT6012 segment (Fig. 3F), prompting us to investigate whether *parA* influences plasmid copy number. Indeed, only the *parA*-expressing strain showed an increase in pKKO3-2 copy number (Fig. 4A). The findings suggest that *parA*-mediated transcriptional activation may be linked to higher plasmid copy number. These qRT-PCR data indicate that ParB likely acts as a transcriptional activator for genes downstream of *parS_NAH_* sequences (*mpfA*, *mpfE*, and *mpfK*), presumably by binding these sites.

### ParA specifically targets the p15A origin to increase plasmid copy number

To determine whether the observed ParA-mediated increase in plasmid copy number was specific to pKKO3-2 or represented a broader effect, we established a quantitative PCR-based system to measure plasmid copy number and applied it to other plasmids. In the presence of *parA*, the copy number of pNIT6012, the parental vector used to construct pKKO3-2, increased significantly (Fig. 4B and C). pNIT6012 is a shuttle vector containing both p15A and pVS1 origins of replication. To identify which replication system was responsible for this increase, we deleted the pVS1-associated replication genes (*repA* and *stb*) (Fig. 2A) and found that this deletion did not affect ParA-mediated copy number elevation, suggesting that the p15A origin is the functional target. Consistently, although to a lesser extent than with pNIT6012, *parA* also increased the copy number of another plasmid (pSTV29) carrying the p15A origin (Fig. 4D). In contrast, plasmids with alternative replication systems such as pSC101 or pBBR1 showed no response to *parA* expression (Fig. 4D). These findings suggest that ParA specifically enhances the replication of p15A-based plasmids.

### Distribution of *parS_NAH_* sites around conjugative transfer and partitioning genes in specific genera

**Fig 5:**
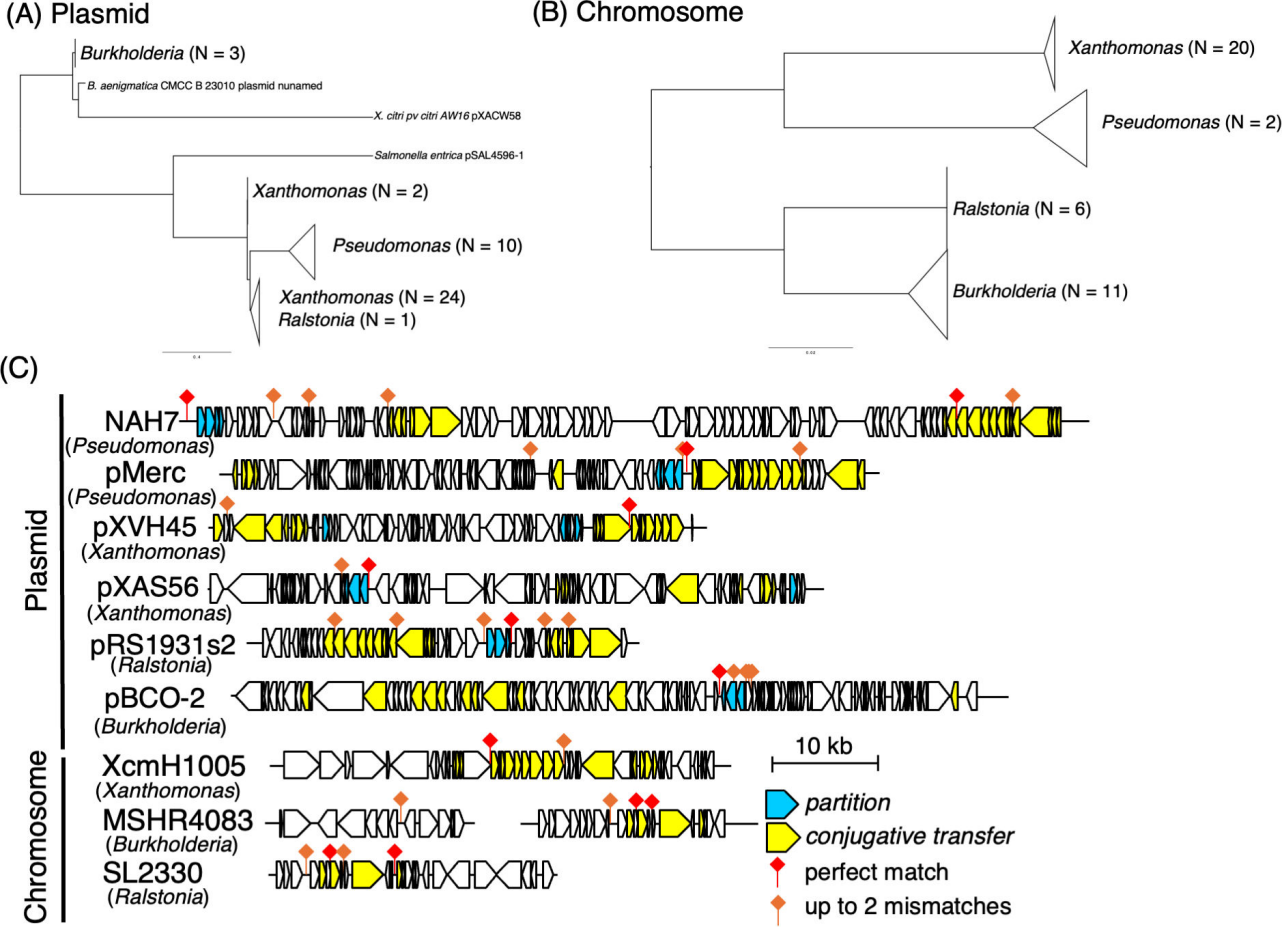
Phylogenetic analysis and distribution of *parS_NAH_* sequences in plasmid and chromosomal sequences. (**A and B**) Phylogenetic trees of sequences containing *parS _NAH_* sites. The sequences with perfect matches to *parS _NAH_* were identified through BLAST searches. The plasmid-associated tree (**A**) was constructed based on amino acid sequences of relaxase proteins, while the chromosomal tree (**B**) was constructed using 16S rRNA gene sequences. Multiple sequence alignments were performed using MAFFT, and phylogenetic trees were visualized with FigTree. In panel **A**, the number of plasmid sequences belonging to each genus is shown in parentheses. In panel **B**, collapsed clades represent groups of chromosomal 16S rRNA sequences from the same genus; the number of sequences within each group is indicated. This analysis highlights the phylogenetic diversity of *parS _NAH_*-associated elements across genera. (**C**) Schematic representation of the genomic context surrounding *parS1_NAH_* sequences and those with up to two nucleotide mismatches in selected plasmids and chromosomal regions. The *parS1_NAH_* sites are indicated by red diamonds (perfect matches) and orange diamonds (up to two mismatches). Genes involved in partitioning (cyan) and conjugative transfer (yellow) are highlighted. The schematic represents the approximate locations of these elements within the 10 kb genomic regions surrounding the *parS1_NAH_* sites. For plasmids, the entire sequence length is represented, while for chromosomes, only the surrounding regions containing the repeat sequences are shown. Specifically, for chromosomal sequences: XcmH1005 (GenBank: CP013004) represents positions 187,208–227,688. MSHR4083 (GenBank: CP017050) represents positions 1,091,874–1,109,185 and 1,954,109–1,971,627. SL2330 (GenBank: CP022794) represents positions 594,289–620,391.

Given that ParB binds to *parS_NAH_* and increases transcription levels downstream of these sites, we hypothesized that these sites might serve as regulatory hubs integrating vertical and horizontal plasmid dissemination. To test this hypothesis, we investigated the conservation and distribution of *parS_NAH_* sequences. We analyzed both plasmids and chromosomes containing at least one perfectly matching *parS1_NAH_* site sequence. As a result, we identified 120 plasmid sequences and 38 chromosomal sequences that contained a perfect *parS1_NAH_* match. For phylogenetic analysis, we constructed a tree using conserved genes from these representative sequences: *relaxase* genes were used for plasmid sequences, whereas 16S rRNA genes were used for chromosomal sequences, enabling classification and visualization by genus (Fig. 5A and B). Plasmid and chromosomal sequences were predominantly associated with genera such as *Pseudomonas*, *Xanthomonas*, *Burkholderia*, and *Ralstonia*. Interestingly, the analyzed *parS_NAH_* site was not only found in the plasmid of NAH7’s original host, *Pseudomonas*, but also in plasmids and chromosomes of several other proteobacterial species, highlighting its broader distribution and potential functional significance.

In addition, we selected representative plasmid and chromosomal sequences to assess the presence of *parS_NAH_* variants. This analysis identified not only perfectly conserved *parS1_NAH_* but also those permitting up to two mismatches. The schematic representation (Fig. 5C) illustrates the distribution of *parS_NAH_* sites, highlighting their positions relative to conserved partitioning and conjugative transfer genes. Importantly, their proximity to partitioning and conjugative transfer genes suggests a potential functional link between these elements and the regulation of plasmid stability and horizontal gene transfer.

### Binding profiles of ParR, TraA, and TraD to the *oriT* region

**Fig 6:**
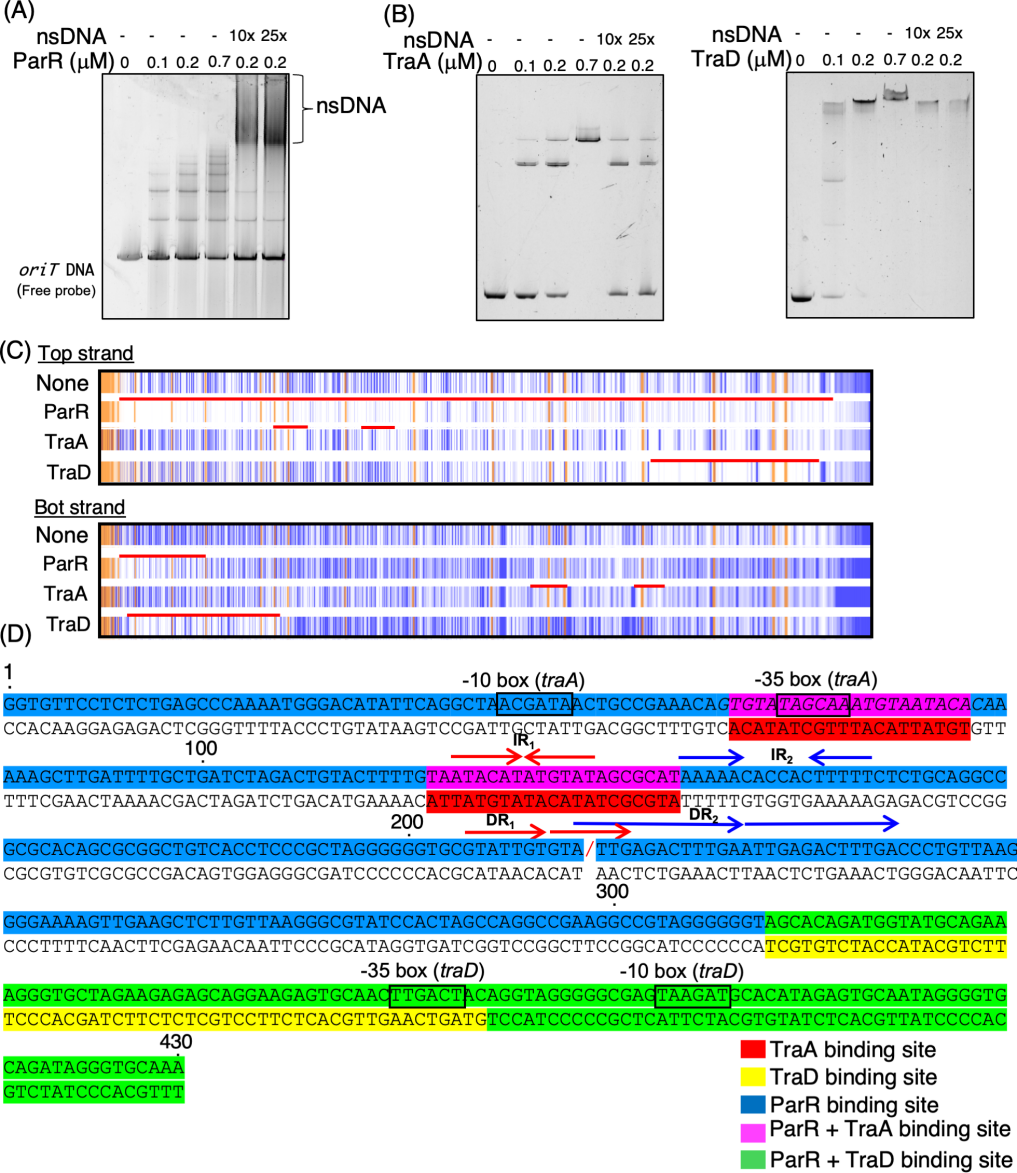
Protein-DNA interactions of ParR, TraA, and TraD with *oriT* DNA. (**A and B**) EMSA demonstrating the binding of ParR-Strep, TraA-His, and TraD-His proteins to *oriT* DNA fragments. (**A**) EMSA using SYBR Gold staining. A 15 nM *oriT* DNA fragment was incubated with ParR-Strep at increasing concentrations (0, 0.1, 0.2, and 0.7 µM), with or without the addition of salmon sperm DNA as a non-specific competitor (10× or 25× relative to the labeled probe). Samples were incubated at 30°C for 5 minutes and resolved on a 15% native polyacrylamide gel in Tris-glycine buffer. The gel was stained with SYBR Gold and visualized using the FLAS-T1500 system. (**B and C**) EMSA using 5′-FAM-labeled oriT DNA probe (45 nM) and TraA-His or TraD-His at indicated concentrations (0, 0.1, 0.2, and 0.7 µM) with or without non-specific competitor DNA (10× or 25×). Gels were imaged directly by detecting FAM fluorescence without post-staining. The lower band represents free (unbound) *oriT* DNA probe. The “*oriT* DNA” represents the free DNA probe. (**C**) DNase I footprinting assay results showing the protection pattern on *oriT* DNA by ParR-Strep, TraA-His, and TraD-His proteins. The assay was conducted using FAM-labeled *oriT* DNA fragments to identify the binding regions on both the top and bottom strands. The orange regions represent internal standard markers (GeneScan 500 LIZ size standard), while the blue lines indicate the FAM-labeled *oriT* DNA fragments obtained after DNase I treatment. DNase I-protected regions indicate protein-DNA interaction sites, with the red line highlighting specific binding regions compared to the “None” control. The data were analyzed using TraceViewer, with corresponding peak data available in Fig. S4 and S5 (**D**) Sequence analysis of the *oriT* region, highlighting the binding sites for ParR, TraA, and TraD proteins. This panel was created based on footprinting analysis (B) and G + A ladder data from Fig. S4 and S5. Specific binding sites are color-coded as follows: ParR binding sites are indicated in blue, TraA binding sites in red, and TraD binding sites in orange. Overlapping binding sites between ParR and TraA are shown in purple, while those shared by ParR and TraD are marked in green. “/” indicates the *nic* site. The -10 and -35 boxes are indicated, representing promoter regions relevant to the transcriptional regulation of conjugative transfer genes. Important IR and DR sequences identified in previous studies as significant for their functions are also annotated.

The role of ParR in activating the transcription of conjugation-related genes has not been supported by experimental evidence. Since ParR contains a KORA domain predicted to bind DNA, its DNA-binding activity likely contributes to the observed increase in conjugation frequency. In the process of conjugative transfer, *oriT* is a particularly important DNA region, serving as the initiation site for DNA transfer. The binding of proteins to this region is believed to play a critical role in facilitating conjugation. Based on this, we investigated whether ParR specifically binds to the *oriT* region to explore its potential role in conjugative transfer (Fig. 6). The EMSA demonstrated that ParR binds to the *oriT* DNA, forming multiple distinct complexes (Fig. 6A). The disappearance of the free probe and the appearance of shifted bands confirm the interaction between ParR and the DNA. Notably, ParR produced a greater number of shifted bands, suggesting the formation of diverse binding complexes.

Homologs of TraA and TraD—specifically TrwA and StbA in the well-studied R388 plasmid—are known to bind the *oriT* region (5, 10). To determine whether TraA and TraD similarly interact with the *oriT* DNA in the NAH7 system, we performed EMSAs (Fig. 6B). Both TraA and TraD bound to the *oriT* region, as indicated by the disappearance of the free probe and the appearance of shifted bands. TraA and TraD displayed a clear binding pattern characterized by discrete, well-defined shifted bands at all tested concentrations. To further assess the DNA-binding specificity of these proteins, EMSA experiments were performed in the presence of an excess amount of non-specific DNA. TraA and TraD maintained their shifted bands even with the addition of nsDNA, indicating strong and specific binding to the *oriT* region. In contrast, ParR showed a partial loss of shifted bands under the same conditions, suggesting that its binding to *oriT* is less specific than that of TraA and TraD.

We further examined ParR’s DNA-binding profile through DNase I footprinting with a FAM-labeled *oriT* DNA fragment. As shown in Fig. 6C, ParR extensively protects the top strand of the *oriT* DNA, including the *nic* site, a key element in conjugative transfer (Fig. 6D). This widespread protection suggests that ParR engages multiple sites along the DNA, possibly through cooperative binding. On the bottom strand, ParR’s protection was more localized, indicating asymmetric binding or varying accessibility between strands. TraA protected the -35 box of the *traABC* gene cluster promoter region and the IR1 sequence (Fig. 6D), which are essential for conjugative transfer (18). TraD, in contrast, protected a broader region encompassing the promoter of the *traDEF* gene cluster, including both the -35 and -10 boxes.

### Functional importance of ParR–DNA interaction in conjugative transfer

**Fig 7:**
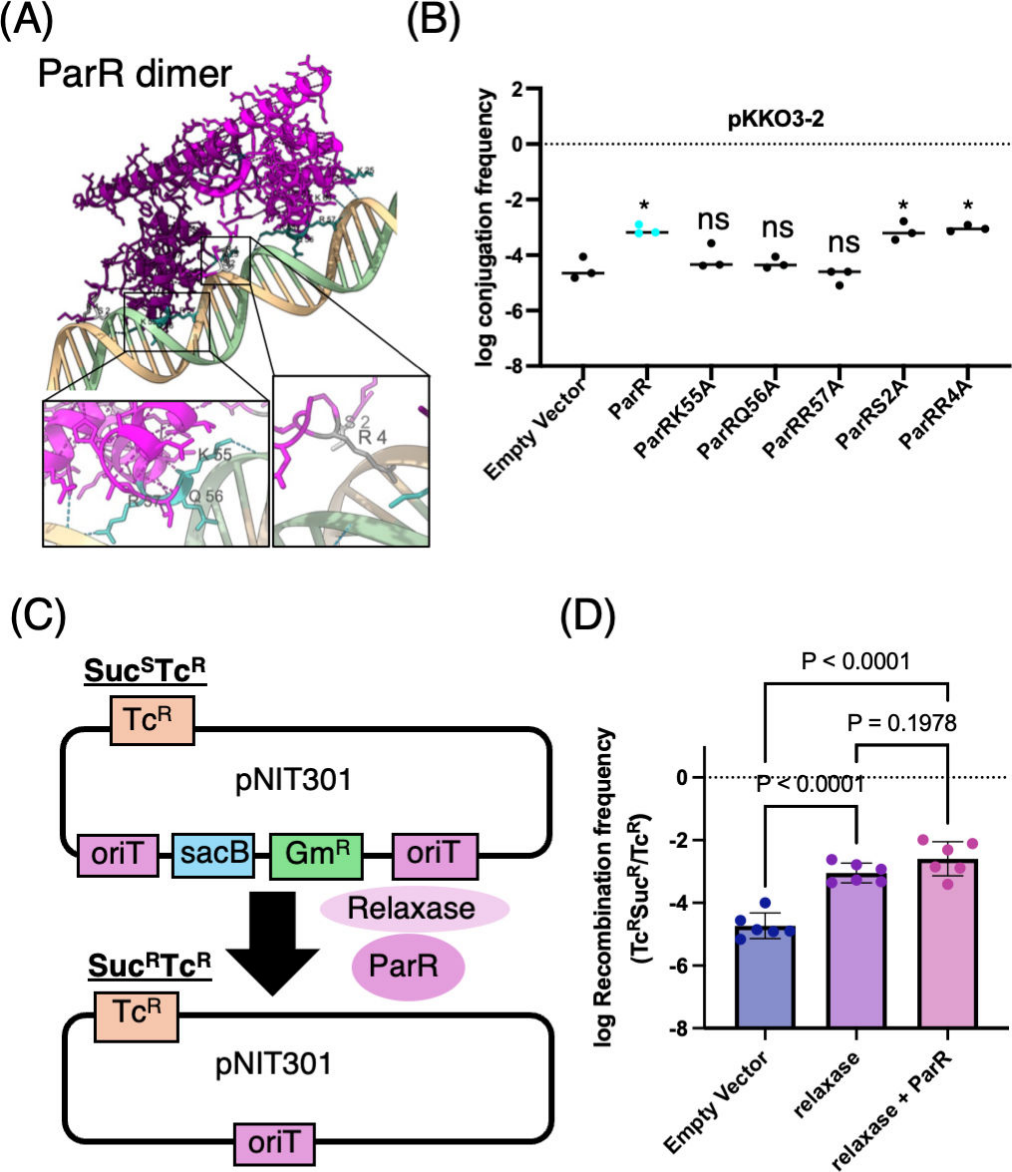
DNA binding by ParR is required for conjugation activation and may assist recombination. (**A**) Predicted structure of the ParR dimer bound to double-stranded *oriT* DNA, generated using AlphaFold3. Close-up views highlight residues K55, Q56, and R57 regions, which are predicted to form hydrogen bonds with DNA, and control residues S2 and R4, which are not involved in direct DNA interaction. (**B**) Effect of point mutations in ParR on conjugative transfer frequency of pKKO3-2. Plasmid transfer assays were performed in *E. coli* expressing wild-type or mutant ParR proteins. Alanine substitutions of K55, Q56, or R57 abolished the enhancement effect, while S2A and R4A mutants retained activity. Statistical significance was determined by one-way ANOVA followed by Dunnett’s multiple comparisons test (*P* < 0.05; ns, not significant). (**C**) Schematic diagram of the site-specific recombination assay using the pNIT301 plasmid, which contains two *oriT* sites flanking the *sacB* and *Gm*^R^ cassettes. Recombination between *oriT* sites excises this region, converting the strain from Suc^S^Tc^R^ to Suc^R^Tc^R^. (**D**) EC100 cells harboring pNIT301 and either pUC18, pUC18­traC, or pKKTH0051 were induced with IPTG to express the respective proteins. After overnight culture, the numbers of tetracycline‐resistant (Tcʳ) and tetracycline-sucrose-resistant (TcSucʳ) colonies were determined. The *oriT–oriT* recombination frequency based on the recovery of Suc^R^ Tc^R^ colonies was quantified. Bars represent means ± SD; *P*-values were determined by one-way ANOVA with Tukey’s test.

To determine whether the enhancement of pKKO3-2 conjugation by ParR depends on its DNA-binding activity, site-directed mutagenesis was performed to disrupt key DNA-interacting residues. AlphaFold3-based structural modeling of the ParR–dsDNA *oriT* complex revealed that residues K55, Q56, and R57 are likely involved in hydrogen bonding with DNA (Fig. 7A and B). Each of these residues was individually substituted with alanine, and none of the resulting ParR variants increased the conjugation frequency, in contrast to the wild-type ParR. In comparison, alanine substitutions of S2 and R4—residues not predicted to interact directly with DNA—did not impair ParR’s ability to enhance conjugation. These results suggest that the DNA-binding activity of ParR is critical for its function in promoting conjugative transfer.

We also investigated whether ParR influences site-specific recombination catalyzed by the relaxase (Fig. 7C and D). To quantify recombination frequency, we used the pNIT301 plasmid, which carries a *sacB* gene flanked by two *oriT* sites and a gentamicin resistance marker. Relaxase expression led to a clear increase in sucrose-resistant colony formation, reflecting higher recombination efficiency between the *oriT* sites. When ParR was co-expressed, a slight but reproducible increase in recombination frequency was observed. These findings suggest that ParR may facilitate relaxase-mediated recombination, possibly by stabilizing DNA interactions at the *oriT* region—and thus play a role in the Dtr system.

## DISCUSSION

**Fig 8:**
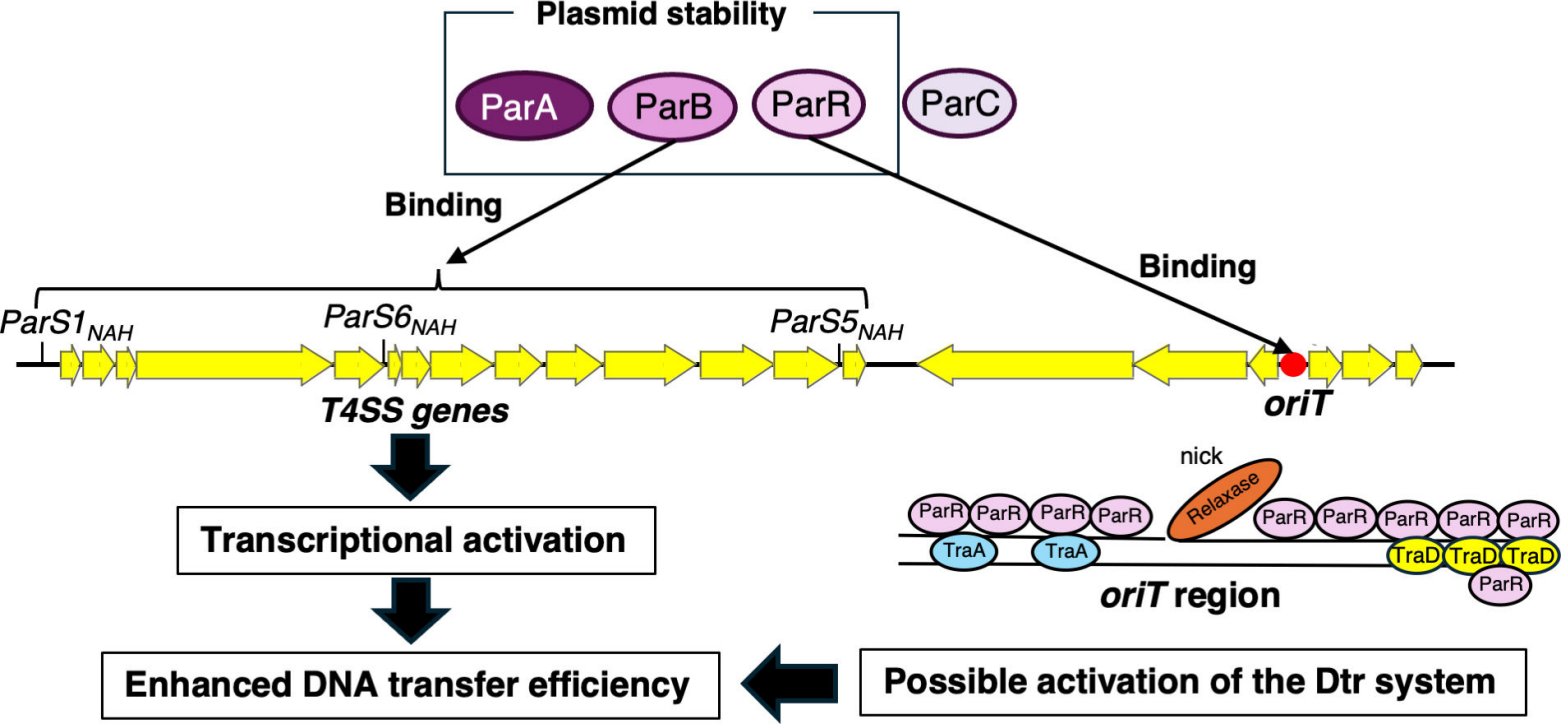
Proposed model of Par proteins in conjugative transfer enhancement in the NAH7 system. This model illustrates the functional roles of ParA, ParB, ParR, and ParC proteins in plasmid stability and conjugative transfer in the NAH7 system. ParA, ParB, and ParR contribute to maintaining plasmid stability within host cells. ParB binds specifically to *parS_NAH_* sites (*parS1_NAH_*, *parS5_NAH_*, and *parS6_NAH_*), which are located near the T4SS genes (yellow arrows). The binding of ParB to *parS* sites results in the transcriptional activation of T4SS-related genes, promoting the assembly of the T4SS and thus enhancing DNA transfer efficiency. At the *oriT* region, ParR binds specifically, potentially aiding in the recruitment of conjugative machinery and interacting with relaxase and T4SS components, such as TraA and TraD. This interaction may help facilitate the initiation of DNA transfer, promoting the Dtr system during conjugation.

As part of elucidating the molecular mechanism of conjugative transfer activation in NAH7, this study investigated the role of the *par* gene cluster, which contributes to plasmid stability (Fig. 1D and Fig. 8), in enhancing conjugative transfer (Fig. 2 and Fig. 8). Our findings demonstrated that ParB acts as a transcriptional activator for genes encoding components of the T4SS, while ParR increases the frequency of conjugative transfer, likely by binding to the *oriT* region, without directly activating the transcription of conjugation-related genes (Fig. 3 and 8). Based on the mechanism of action of ParB and ParR in enhancing the conjugative transfer frequency of pKKO3-2, it is that these proteins also enhance the conjugative transfer frequency of the native NAH7 plasmid. In NAH7-harboring strains, a slight increase in conjugative transfer frequency was observed upon the expression of either *parB* or *parR* (Fig. S6). However, the presence of *par* genes on NAH7 may mask the frequency increase, making it challenging to detect further enhancements. In contrast, ParA seems to enhance conjugative transfer by increasing the copy number of the original vector, pNIT6012 (Fig. 4B and C). This increase in copy number likely leads to elevated transcription levels of conjugation-related genes, resulting in a higher frequency of conjugative transfer. No evidence was found to support a direct role for ParA in enhancing the conjugative transfer frequency of the native NAH7 plasmid.

### Biological significance of *par* genes in conjugative transfer enhancement

Since plasmid partitioning is synchronized with host cell division, under nutrient-rich and favorable conditions that support host cell division, probably NAH7 not only efficiently distributes to daughter cells but also activates conjugative transfer, enabling its spread to other bacterial cells. This dual function may represent an adaptive strategy for the plasmid, as its original host, *P. putida* G7, typically inhabits nutrient-poor soil environments (29). The ability to transfer to other cells under nutrient-rich conditions could provide a significant survival advantage for the plasmid.

### Mechanisms of enhanced conjugative transfer by ParR and ParB in NAH7: comparison with KorA and KorB in RP4

In the IncP-1α plasmid RP4, the CBP KorB acts as a transcriptional repressor, specifically targeting the *trbB* promoter, and this repressive action is further enhanced by cooperative interactions with another repressor, TrbA (30). In contrast, our study demonstrated that ParB binds to the *parS_NAH_* sites and promotes the transcription of conjugation-related genes (*mpfA*, *mpfE*, and *mpfK*) located downstream of this site. The similarity between ParB and KorB is low, particularly in the DNA-binding domain (Fig. S7B). Although both the *parS* site in NAH7 and the OB sites in RP4 are IR sequences (Fig. S7A), their lengths and sequences differ significantly, reflecting their distinct functional roles—transcriptional activation by ParB versus repression by KorB. Additionally, KorB is known to work cooperatively with other DNA-binding proteins such as KorA to achieve effective transcriptional repression (31). While it remains unclear whether ParB also collaborates with other regulatory factors, our findings indicate a potential cooperative interaction between ParB and ParR. Co-expression of ParB and ParR led to a higher conjugative transfer frequency of pKKO3-2 compared to the expression of either protein alone.

KorA, a homolog of ParR, is known to function as a transcriptional repressor. However, no evidence supports that ParR regulates conjugation-related genes. Instead, EMSA and DNase I footprinting assays revealed that ParR binds to multiple sites on the *oriT* DNA with varying affinities or forms oligomers with different stoichiometries. The ParR binding to *oriT* DNA may play a role in facilitating its function in conjugative transfer. ParR slightly enhanced the site-specific recombination activity catalyzed by the relaxase between two *oriT* sites. It is also possible that ParR, in interactions with the relaxase protein (Fig. S8), contributes to the assembly of the relaxosome, thereby enhancing the efficiency of conjugative transfer. ParR likely has functions beyond binding to *oriT*, as the disruption of the *parR* gene led to reduced plasmid stability even in the absence of *oriT* (e.g., in pK7OR). This suggests that ParR contributes to plasmid stability through *oriT*-independent mechanisms.

### Unexpected increase in plasmid copy number induced by ParA

Shuttle vectors carrying both p15A and pVS1 origins of replication, such as pNIT6012, are widely used for molecular cloning and gene expression in multiple bacterial hosts (32). In this study, we demonstrated that ParA from the NAH7 specifically acts on the p15A origin to increase plasmid copy number. ParA expression also elevated the copy number of pSTV29, another plasmid harboring the p15A origin. Interestingly, while ParA expression led to a slight growth defect in cells carrying pSTV29, no such defect was observed in cells harboring pNIT6012, suggesting vector-specific differences in the cellular burden of copy number amplification. The p15A origin regulates plasmid copy number through an antisense RNA mechanism. Specifically, transcription of RNA II generates a primer for DNA replication, while a shorter antisense RNA, RNA I, inhibits primer formation by forming a stable duplex with RNA II. The Rom (Rop) protein further stabilizes this interaction, fine-tuning replication initiation. Given this regulation system, it is plausible that ParA interferes with the RNA I–RNA II interaction or modulates the accessibility or processing of RNA II, thereby enhancing initiation events and ultimately increasing plasmid copy number. The ability to control plasmid copy number is considered valuable as a genetic tool (33, 34), especially since plasmids transferred through conjugation play a crucial role in genetic engineering and biotechnology.

### Comparison with other conjugative transfer enhancement mechanisms

Mechanisms involving the enhancement of conjugative transfer by partitioning proteins have also been reported in other plasmids, such as plasmid R1 and the Ti plasmid (15, 35). In plasmid R1, the Type II partition system ATPase ParM_R1_ and the centromere-binding protein ParR_R1_ interact directly with components of the conjugative machinery, thereby enhancing DNA transfer efficiency. Specifically, ParM_R1_ and ParR_R1_ physically and functionally interact with the relaxase, stimulating its relaxase activity, which is essential for initiating DNA transfer. Additionally, ParM_R1_ interacts with T4SS ATPases such as TraD _R1_ (VirD4) and TraC _R1_ (VirB4), indicating a coordinated role in facilitating the assembly and function of the T4SS. In contrast, in the NAH7 system, we found that conjugative transfer frequency increases through the binding of ParB or ParR to DNA, while the involvement of direct protein-protein interactions remains unclear. Using the AlphaFold3 server, we predicted the interactions between six protein pairs: dimer ParR with TraC (relaxase), TraB (VirD4), and MpfC (VirB4). All interface predicted TM scores, which are used to evaluate the confidence of predicted relative positions of subunits forming a protein-protein complex, were obtained, with the highest interaction scores observed for ParR-MpfC dimer, ParR-TraB dimer, and ParR-TraC dimer (Fig. S8). It is possible that ParR enhances conjugation frequency not only by binding to the *oriT* region but also by interacting with other components of the conjugative transfer system. Although NAH7 and R1 possess different types of partitioning systems, it is likely that NAH7 utilizes a mechanism similar to R1 involving direct protein-protein interactions for enhancing conjugative transfer frequency.

## MATERIALS AND METHODS

### Strains and growth conditions

*E. coli* strains listed in Table 1 were grown in Lysogeny Broth (LB). *P. putida* KT2440 strains were grown in one-third LB media for plasmid stability assay at 30°C. Strains and plasmids were maintained with appropriate antibiotic selection: ampicillin (100 µg/mL), tetracycline (20 µg/mL), kanamycin (50 µg/mL), gentamycin (10 µg/mL), and rifampicin (100 µg/mL).

**TABLE 1:** Strains and plasmids used in this study

Strain or plasmid	Relevant characteristics	Source or reference
*E. coli* DH5alpha	*F^-^ recA1 endA1 gyrA96 thi-1 hsdR17 supE44 relA1 ∆(lacZYA-argF) Φ80lacZDM15*	(36)
*E. coli* EC100	*mcrA Δ(mrr-hsdRMS-mcrBC*) f80d*lacZΔM15 ΔlacX74 recA1 endA1 araD139 Δ(ara-leu)7697 galU galK rpsL nupG*	Epicentre, Inc.
*E. coli* EC100Rif	Rif^R^ mutant of EC100	This study
*E. coli* BL21(DE3)	*F^-^ ompT hsdSB (rB^-^mB^-^) gal, lambda* (DE3)	(37)
*Pseudomonas putida* KT2440	Wild-type strain	ATCC 470554
Plasmids		
NAH7K2	NAH7Δ*nahAc*; Km^r^	(38)
pNIT6012	p15a ori and pVS1 ori; shuttle vector, Tc^r^	(25)
pKKO3	pKKO2Δ(*orf30–33*)::Km^r^	(19)
pNITara	Tc^r^; pNIT6012 derivative carrying araC gene and pBAD promoter	(39)
pKKO3-2	pNIT6012 derivative carrying *traF–traC*, *mpfK–mpfR*	This study
pK18mob	Km^r^; *E. coli* pMB1 vector	(40)
pK7OR	pK18mob derivative carrying NAH7 replication system (*parS1_NAH_–rep* region)	This study
pKKTH0026	pK7OR∆*parA*	This study
pKKTH0027	pK7OR∆*parB*	This study
pKKTH0028	pK7OR∆*parR*	This study
pKKTH0029	pK7OR∆*parC*	This study
pKKTH0116	pK7OR∆*parA-parC*	This study
pKKTH0099	pK7OR derivative: internal deletion of amino acids 15-214 in *parA*	This study
pKKTH0100	pK7OR derivative: internal deletion of amino acids 35-232 in *parB*	This study
pKKTH0101	pK7OR derivative: internal deletion of amino acids 35-79 in *parR*	This study
pKKTH0102	pK7OR derivative: 2 bp deletion at codon 44, causing frameshift in *parA*	This study
pKKTH0103	pK7OR derivative: 2 bp deletion at codon 68, causing frameshift in *parB*	This study
pKKTH0104	pK7OR derivative: 2 bp deletion at codon 42, causing frameshift in *parR*	This study
pKKTH0046	pNITara derivative carrying *parA* gene	This study
pKKTH0047	pNITara derivative carrying *parB* gene	This study
pKKTH0048	pNITara derivative carrying *parR* gene	This study
pUC18	Ap^r^; *E. coli* vector	(41)
pUC18KU1	pUC18 derivative carrying *parA* gene	This study
pUC18KU2	pUC18 derivative carrying *parA, parB, parR, parC* genes	This study
pUC18KU8	pUC18 derivative carrying *parR* gene	This study
pUC18KU9	pUC18 derivative carrying *parC* gene	This study
pUC18KU10	pUC18 derivative carrying *parB* gene	This study
pKKTH0019	pUC18 derivative carrying *parA, parB* genes	This study
pKKTH0020	pUC18 derivative carrying *parA, parC* genes	This study
pKKTH0021	pUC18 derivative carrying *parB, parR* genes	This study
pKKTH0022	pUC18 derivative carrying *parB, parC* genes	This study
pKKTH0023	pUC18 derivative carrying *parR, parC* genes	This study
pKKTH0030	pUC18 derivative carrying *parA, parR* genes	This study
pUC18KU4	pUC18 derivative carrying *parA, parR, parC* genes	This study
pUC18KU5	pUC18 derivative carrying *parA, parB, parC* genes	This study
pUC18KU6	pUC18 derivative carrying *parA, parB, parR* genes	This study
pUC18KU7	pUC18 derivative carrying p*arB, parR, parC* genes	This study
pKKTH0082	pET22b derivative carrying *parR*-strep-tag gene	This study
pKKTH0105	pUC18 derivative expressing ParR with serine 2 replaced by alanine	This study
pKKTH0106	pUC18 derivative expressing ParR with serine 3 replaced by alanine	This study
pKKTH0107	pUC18 derivative expressing ParR with arginine 4 replaced by alanine	This study
pKKTH0109	pUC18 derivative expressing ParR with lysine 55 replaced by alanine	This study
pKKTH0110	pUC18 derivative expressing ParR with glutamine 56 replaced by alanine	This study
pKKTH0111	pUC18 derivative expressing ParR with threonine 57 replaced by alanine	This study
pKKTH0008	pET22b derivative carrying *parB*-Hisx6 gene	This study
pKKTH0061	pET22b derivative carrying *traD*-Hisx6 gene	This study
pKKTH0062	pET22b derivative carrying *traA*-Hisx6 gene	This study
pNIT101	pNIT6012 derivative carrying *oriT*_NAH7	(18)
pNIT301	pNIT6012 derivative carrying sacB and Gm^r^ genes flanked by two copies of *oriT* NAH7 region	(18)
pUC18traC	pUC18 derivative carrying NAH7 *traC* gene	(18)
pKKTH0051	pUC18 derivative carrying *traC, parR* genes	This study
pUC18mpfKup	pUC18 derivative carrying *parS_NAH_5*	This study
pKKTH0114	pNIT6012∆*rep*	This study
pKKTH0115	pNIT6012∆*stb*	This study
pSTV29	Bacterial cloning vector with a p15A origin and a chloramphenicol resistance gene	Takara
pMW216	Bacterial cloning vector with a pSC101 origin and a kanamycin resistance gene	NIPPON GENE CO.
pBBR1MCS-2	Bacterial cloning vector with a pBBR origin and a kanamycin resistance gene	GenBank: U23751.1

### Plasmid construction

All plasmids and oligonucleotide primers used in these studies are listed in Table 1 and Table S1. pKKO3-2 is a plasmid derived from the self-transmissible plasmid pKKO3 (19), with the Km resistance gene removed. The Km resistance gene on pKKO3 is flanked by KpnI sites, allowing it to be excised by KpnI digestion, followed by self-ligation. The removal of the Km resistance gene was confirmed by PCR and by the loss of Km resistance.

pK7OR is a mini-NAH7 plasmid containing the region from *parS1_NAH_* to the *rep* gene. A DNA fragment containing this region was amplified from NAH7, digested with XhoI and SpeI, and inserted into the SalI-XbaI site of pK18mob (40). Plasmids pKKTH0026 to pKKTH0029 are derivatives of pK7OR, each lacking one of the *par* genes. To construct these plasmids, DNA fragments with specific *par* gene deletions were amplified from pK7OR and self-ligated using the Gibson Assembly kit (New England Biolabs, Beverly, MA, USA). pKKTH0116 was constructed by digesting pK7OR with HindIII, followed by self-ligation. Internal deletion mutants of each par gene were constructed as follows: plasmids pKKTH0099 (parAΔ15-214), pKKTH0100 (parBΔ35-232), and pKKTH0101 (parRΔ35-79) were generated by PCR amplification of pK7OR using primer pairs oligoNucKK0492–0493, oligoNucKK0494–0495, and oligoNucKK0496–0497, respectively, followed by self-ligation of the PCR products. Frameshift mutants of the par genes were similarly constructed using pK7OR as the template. Plasmids pKKTH0102 (parA∆fs), pKKTH0103 (parB∆fs), and pKKTH0104 (parR∆fs) were obtained by PCR using primer pairs oligoNucKK0498–0499, oligoNucKK0500–0501, and oligoNucKK0502–0503, followed by self-ligation. The precise deletion and frameshift positions for each construct are shown in Fig. S1. pKKTH0046, pKKTH0047, and pKKTH0048, each expressing a single *par* gene, were constructed by amplifying individual *par* gene regions from NAH7 and cloning them into the KpnI site of pNITara using the Gibson Assembly kit.

pUC18KU1, pUC18KU8, pUC18KU9, and pUC18KU10, each expressing a single *par* gene, were constructed by amplifying individual *par* gene regions from NAH7 and cloning them into the KpnI site of pUC18 (41) using the Gibson Assembly kit. Plasmid pUC18KU2, which expresses all four *par* genes, was similarly constructed by amplifying the entire *par* gene region from NAH7 and cloning it into the KpnI site of pUC18 using the Gibson Assembly kit.

Plasmids expressing combinations of two *par* genes were constructed by amplifying DNA fragments from specific templates and cloning each fragment into the KpnI site of pUC18 using the Gibson Assembly Kit. For pKKTH0019, pKKTH0021, pKKTH0022, pKKTH0023, and pKKTH0030, the respective templates used were pUC18KU2, pUC18KU2, pUC18KU5, pUC18KU2, and pUC18KU4. Plasmid pKKTH0020 was constructed by amplifying a fragment from pUC18KU4, phosphorylating it, and performing self-ligation.

Plasmids expressing combinations of three *par* genes were constructed by amplifying specific gene regions from NAH7 and cloning each combination into the KpnI site of pUC18 using the Gibson Assembly Kit. For pUC18KU6 and pUC18KU7, the *parA-parR* and *parB-parC* regions were amplified with primer sets KUDO011-KUDO29 and KUDO012-KUDO038, respectively. For pUC18KU4 and pUC18KU5, the *parA* and *parRC* regions, and the *parAB* and *parC* regions, were amplified using primer sets KUDO011-KUDO026, KUDO012-KUDO025, KUDO011-KUDO028, and KUDO012-KUDO027, respectively. Each construct was designed to be IPTG-inducible.

Plasmids pKKTH0082, pKKTH0008, pKKTH0061, and pKKTH0062 were constructed to express affinity-tagged proteins. DNA fragments of each gene region were amplified and cloned into the NdeI and XhoI sites of pET22b using the Gibson Assembly Kit. For pKKTH0082, which expresses ParR-strep, the oligonucleotide primer (oligoNucKK0319) included a sequence for the Strep-tag. Point-mutant parR expression plasmids pKKTH0105, pKKTH0106, pKKTH0107, pKKTH0109, pKKTH0110, and pKKTH0111 were generated by PCR amplification using pUC18KU8 as the template and primer pairs listed in Table S2, followed by self-ligation of the PCR products. Plasmid pKKTH0051 was constructed by cloning parR downstream of *traC* into the NdeI site of pUC18traC in the same transcriptional orientation. The plasmid pUC18mpfKup, containing the upstream *parS5_NAH_* region of *mpfK*, was constructed by annealing two oligonucleotides, Bam_up_orf34_kpn and Bam_up_orf34_kpn_COM, to create a double-stranded DNA fragment. This fragment was then digested with BamHI and KpnI and ligated into the corresponding sites of pUC18. pKKTH0114 and pKKTH0115 were constructed by PCR amplification of pNIT6012, excluding the *repA* or *stb* region, respectively, followed by self-ligation of the amplified fragments.

### Plasmid stability assay

pK7OR and its derivative plasmids, which contain the Km resistance gene, were tested for stability using *P. putida* KT2440 as the host strain. The strains were first grown overnight in one-third LB medium supplemented with kanamycin. Subsequently, cultures were diluted 0.1% into fresh one-third LB medium without antibiotics and incubated for 24 hours (approximately 10 generations). Complemented strains were constructed by introducing plasmids carrying each *par* gene under the control of the pBAD promoter into the corresponding mutant backgrounds. Arabinose was added at a final concentration of 10 mM to induce gene expression. After incubation, the cultures were plated onto one-third LB agar plates without antibiotics. On the following day, 100 colonies were randomly picked and streaked onto one-third LB agar plates with kanamycin. The proportion of Km-resistant colonies was then calculated. The assay was performed in three independent biological replicates. The average values were plotted with individual data points, and standard deviations are represented as error bars.

### Conjugation assay

Donor and recipient cells were grown overnight at 30°C in the presence of appropriate antibiotics. Each culture (1 mL) was harvested by centrifugation, and the cell pellets were resuspended in 50 µL of fresh medium. Donor and recipient suspensions were then mixed at a 1:1 cell ratio to a final volume of 100 µL, spotted onto LB agar plates supplemented with 0.5 mM IPTG for induction, and incubated for 6 hours at 30°C. Following incubation, cells were resuspended in 1 mL of LB, serially diluted in LB, and plated on LB agar containing antibiotics to select for transconjugant (Tc), recipients, and donors (D). Plates were incubated overnight at 30°C. DNA transfer frequency is reported as the number of transconjugants per donor (Tc/D). All mating experiments were performed at least three times in triplicate, and representative data are shown with individual data points, average transfer frequencies as bars, and standard deviations as error bars.

### Quantification of the expression of conjugative transfer genes in pKKO3-2

RNA extraction samples were prepared as follows: pKKO3-2 strains expressing *par* genes from pUC18 were each grown overnight, and 1 mL of each culture was centrifuged to harvest the cells. The cell pellets were resuspended in 50 µL of fresh medium. This culture was spotted onto LB agar plates containing 0.5 mM IPTG and incubated at 30°C for 3 hours. After incubation, the cells were resuspended in LB medium, and RNA was extracted from these samples using RNeasy (Qiagen, Hilden, Germany) following the manufacturer’s protocol.

After extraction, each RNA sample was treated with DNase I (Takara, Shiga, Japan) at 37°C for 2 hours to remove residual DNA. Reverse transcription was performed with ReverTra Ace qPCR RT Master Mix (Toyobo, Osaka, Japan) and random 9-mer primers. qRT-PCR was conducted using Luna Universal qPCR Master Mix (New England Biolabs, Beverly, MA, USA) on a CFX Connect Real-Time System (Bio-Rad Laboratories, Hercules, CA, USA). The PCR protocol included an initial denaturation at 95°C for 1 minute, followed by 40 cycles of 10 seconds at 95°C, 10 seconds at 60.0°C, and 10 seconds at 68°C. The primer sets oligoNucKK0074-oligoNucKK0075, oligoNucKK0076-oligoNucKK0077, oligoNucKK0078-oligoNucKK0079, oligoNucKK0080-oligoNucKK0081, oligoNucKK0082-oligoNucKK0083, oligoNucKK0106-oligoNucKK0107, and oligoNucKK0084-oligoNucKK0085 were used to quantify *mpfA*, *mpfE*, *mpfK*, *traD*, *traB*, *tetA,* and *gyrA*, respectively. Expression levels of each target gene were normalized to the expression level of the *gyrA* gene on the *E. coli* chromosome. Individual data points and average expression levels are represented as bars, and standard deviations are shown as error bars.

### Electrophoretic mobility shift assay

ParB-His, TraA-His, TraD-His, and ParR-Strep were expressed in BL21(DE3) cells containing the expression plasmids pKKTH0008, pKKTH0062, pKKTH0061, and pKKTH0082, respectively. Cells were cultured at 30°C, and protein expression was induced at an OD_660_ of approximately 0.5 by adding 0.5 mM IPTG. After 6 hours of induction, cells were harvested and resuspended in RIPA buffer (50 mM Tris-HCl [pH 8.0], 150 mM NaCl, 1% [vol/vol] Triton X-100, 1% [wt/vol] sodium deoxycholate, and 0.1% [wt/vol] SDS). Following sonication, the supernatant was used for protein purification. His-tagged proteins were purified using Ni-NTA agarose (Qiagen, Hilden, Germany), and the Strep-tagged protein was purified using the Strep-Tactin XT 4Flow Starter Kit (IBA, Göttingen, Germany). The purity of each protein was confirmed by SDS-PAGE and CBB staining (Fig. S9).

The purified proteins, TraA-His, TraD-His, and ParR-Strep, were incubated with a FAM-labeled oriT DNA fragment in a binding buffer containing 100 mM NaCl, 80 mM Tris-HCl (pH 7.5), 0.1 mM EDTA, and 5 mM MgCl_2_ at 30°C for 5 minutes. For TraA-His and TraD-His, the DNA concentration was 45 nM, whereas for ParR-Strep, it was 15 nM. Final protein concentrations of 0, 0.1, 0.2, and 0.7 µM were prepared for these experiments. Salmon sperm DNA was added as a non-specific competitor at 10- and 25-fold molar excess relative to the labeled DNA. For ParB-His, a different binding buffer was used, composed of 50 mM Tris-HCl (pH 7.5), 10 mM MgCl_2_, 1 mM ATP, and 10 mM DTT. The ParB-His protein was incubated with FAM-labeled DNA corresponding to the upstream *parS5_NAH_* region of *mpfK* at a final DNA concentration of 82 nM at 30°C for 15 minutes. The final concentrations of ParB-His were 0, 0.1, 0.2, and 0.7 µM. Salmon sperm DNA was added as a non-specific competitor at 10- and 25-fold molar excess relative to the labeled DNA. Samples were electrophoresed on a 15% polyacrylamide gel at 200 V for 90 minutes using Tris-glycine buffer. For TraA, TraD, and ParB, the FAM-labeled DNA was directly detected using the FLAS-T1500 system (Fujifilm, Tokyo, Japan). For ParR, the gel was stained with SYBR Gold (Invitrogen, Carlsbad, CA, USA) prior to detection with the same system.

### Quantification of plasmid copy number by quantitative PCR

To quantify plasmid copy number, *E. coli* EC100 strains harboring each test plasmid were cultured overnight at 30°C in LB medium containing the appropriate antibiotics. When necessary, glucose was added to the medium to suppress *parA* expression from the lac promoter. After incubation, 1 mL of culture was collected and concentrated to approximately 50 µL. The concentrated culture was then spotted onto LB agar plates supplemented with 0.5 mM IPTG and incubated at 30°C for 3 hours to induce *parA* expression. Following induction, the cells were resuspended in sterile water and lysed by boiling at 95°C for 5 minutes. The lysates were centrifuged to remove cell debris, and the resulting supernatants were used as DNA templates for quantitative PCR. The chromosomal gene *gyrA* was used as an internal reference, and plasmid-specific primers were used to amplify target sequences from each plasmid. Relative plasmid copy numbers were calculated by comparing Ct values of *parA*-expressing strains with those of control strains carrying the empty vector.

### DNase I footprinting assay

Two types of FAM-labeled *oriT* DNA fragments were amplified using pNIT101 as the template with primer sets pNIT5041_FAM-pNIT5548 and pNIT5041-pNIT5548_FAM, ensuring that each fragment was labeled on a single strand. The purified proteins TraA-His and TraD-His were incubated with the *oriT* DNA fragment (12 nM) at a final concentration of 1 µM each, and ParR-Strep was incubated at a final concentration of 8 µM. The binding reactions were performed in a buffer containing 100 mM NaCl, 80 mM Tris-HCl (pH 7.5), 0.1 mM EDTA, 5 mM MgCl_2_, and 50 µg/mL salmon sperm DNA at 30°C for 15 minutes.

Following the incubation, the reaction mixtures were treated with 1/10 unit of DNase I at 12°C for 1 minute. The reactions were stopped by heating at 98°C for 10 minutes to inactivate DNase I. The DNase I-treated fragments were purified using the BigDye XTerminator purification kit (Thermo Fisher Scientific).

The purified products were mixed with the GeneScan 500 LIZ size standard (Life Technologies) and separated using an ABI Prism 3130xl Genetic Analyzer. To prepare a G + A sequencing ladder sample, the method of Eckert (42) was used, treating the FAM-labeled dsDNA fragments with formic acid and piperidine. The resulting data were analyzed using the TraceViewer software (43). The raw data supporting these findings are presented in Fig. S4 and S5.

### Resolution assay

The frequency of intramolecular recombination between two *oriT* sites on the plasmid pNIT301 was examined in the *E. coli* strain EC100. Plasmid pNIT301 was introduced into EC100, EC100(pUC18), EC100(pUC18-traC), or EC100(pKKTH0051) by transformation, and transformants were selected on LB agar containing gentamicin (Gm). Each Gm^R^ transformant was then cultured overnight in LB broth supplemented with tetracycline (Tc). The resulting cultures were plated on two types of agar: LB agar lacking NaCl and containing Tc, and LB agar lacking NaCl and containing both Tc and 10% sucrose. Recombination between the *oriT* sites leads to excision of the *sacB* cassette, rendering the cells resistant to sucrose. Therefore, the recombination frequency was calculated as the number of colonies resistant to both Tc and sucrose divided by the number of Tc-resistant colonies.

### Bioinformatics analysis

To estimate the functions of the Par proteins from the NAH7 plasmid (Fig. S1), the amino acid sequences were used as queries for InterPro analysis to identify conserved domains and predict functional annotations. To obtain predicted structures of these Par proteins, we utilized the AlphaFold Database (44) and AlphaFold Server (45) for structure prediction.

To assess the conservation of ParABRC homologs of NAH7 within IncP-9 plasmids (17), a BLASTP analysis was conducted using the protein sequences of IncP-9 plasmids as a database. Each of NAH7’s ParA, ParB, ParR, and ParC proteins was used as a query. An E-value threshold of 10^−8^ was applied to determine significance. Hits meeting this criterion were marked as “present,” whereas sequences without significant hits were labeled as “absent.” The results are summarized in Table S2.

To identify plasmid and chromosomal sequences that perfectly match the NAH7 *parS1* site (18 bp sequence: 5′-TTTCTCGCATGCGAGAAA-3′), we used the NCBI Dataset tool to filter for “complete plasmid” and “complete genomes” data sets. The filtered sequences were then subjected to BLASTN searches to detect the presence of the *parS1_NAH_* site across different bacterial genomes. To classify the identified sequences phylogenetically, we utilized the amino acid sequences of plasmid-encoded relaxase genes and the DNA sequences of 16S rRNA genes from chromosomes. Detection of relaxase genes was performed by conducting a BLASTP search against the experimentally verified relaxase sequences obtained from oriTDB (46), with an *e*-value cutoff of 1e-5. If multiple plasmids carried identical relaxase genes, we selected a single representative sequence from among them for all subsequent analyses. The 16S rRNA gene sequences were extracted from GenBank by selecting annotated 16S rRNA entries. The detected sequences were aligned using MAFFT (47) with the NJ method, and a phylogenetic tree was constructed to visualize the relationships among the sequences. The resulting tree was modified for better visualization using FigTree (http://tree.bio.ed.ac.uk/software/figtree/).

From representative sequences, EMBOSS: fuzznuc was used to find the *parS1_NAH_* site that perfectly matched or had up to two mismatches. The approximate positions of these sites were plotted on gene maps in PowerPoint for visual representation.

Protein structure prediction was performed using the AlphaFold Protein Structure Database server (https://alphafoldserver.com/). The resulting structural models were visualized and prepared using UCSF ChimeraX (48).

### Statistical analysis

All statistical analyses were performed using GraphPad Prism 9. Ordinary one-way ANOVA with Dunnett’s multiple comparisons test or unpaired two-tailed *t*-tests were used as specified in the figure legends. *P*-values and statistical significance are indicated in the figures.

## SUPPLEMENTAL MATERIAL

Fig. S1 mbio.01600-25-s0001.tiff Domain architecture and engineered mutation sites in Par proteins encoded on plasmid NAH7.

Fig. S2 mbio.01600-25-s0002.tiff Effects of internal deletions, frameshift mutations, and complementation on plasmid stability.

Fig. S3 mbio.01600-25-s0003.tiff Inability to isolate a pure NAH7Δ*parABRC*::KmR mutant.

Fig. S4 mbio.01600-25-s0004.tiff DNase I footprinting analysis of the *oriT* region on the top strand.

Fig. S5 mbio.01600-25-s0005.tiff DNase I footprinting analysis of the *oriT* region on the bottom strand.

Fig. S6 mbio.01600-25-s0006.tiff Conjugative transfer frequency of NAH7 in strains expressing Par proteins.

Fig. S7 mbio.01600-25-s0007.tiff Sequence comparison of ParB_NAH7 with KorB_RP4 and ParR_NAH7 with KorA_RP4.

Fig. S8 mbio.01600-25-s0008.tiff Structural predictions of ParR dimer interactions with conjugation-related proteins using AlphaFold3.

Fig. S9 mbio.01600-25-s0009.tiff SDS-PAGE analysis of purified proteins.

Supplemental Tables mbio.01600-25-s0010.xlsx Tables S1 and S2.

ASM does not own the copyrights to Supplemental Material that may be linked to, or accessed through, an article. The authors have granted ASM a non-exclusive, world-wide license to publish the Supplemental Material files. Please contact the corresponding author directly for reuse.
