# Supplementary figures and images for: Type I partition-related proteins enhance conjugative transfer through transcriptional activation and oriT region binding
Source: mBio. 2025 Jul 31;16(9):e01600-25. doi: 10.1128/mbio.01600-25 (PMC12421886; doi:10.1128/mbio.01600-25)

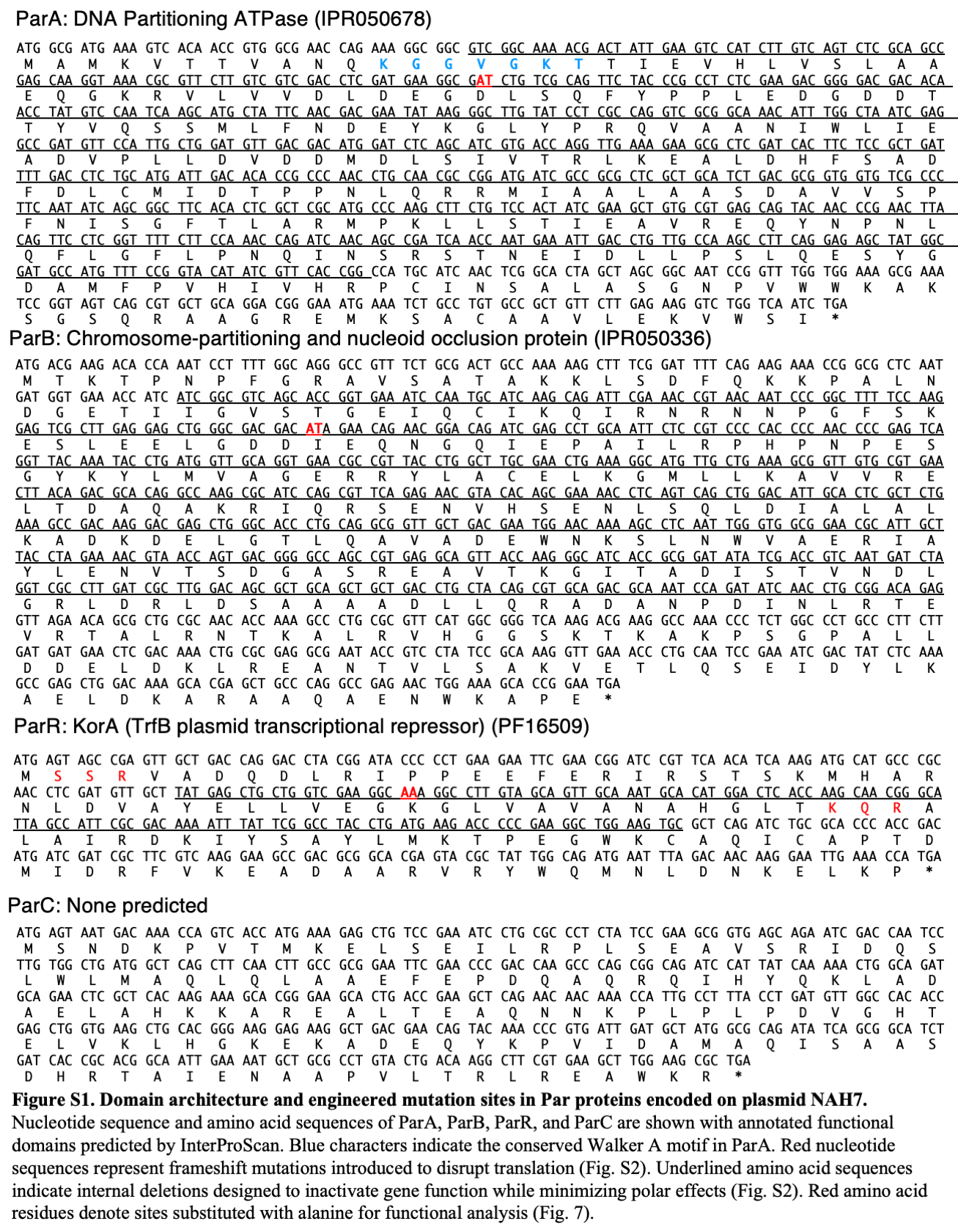

Supplement: Fig. S1 — Domain architecture and engineered mutation sites in Par proteins encoded on plasmid NAH7. [file mbio.01600-25-s0001.tiff]

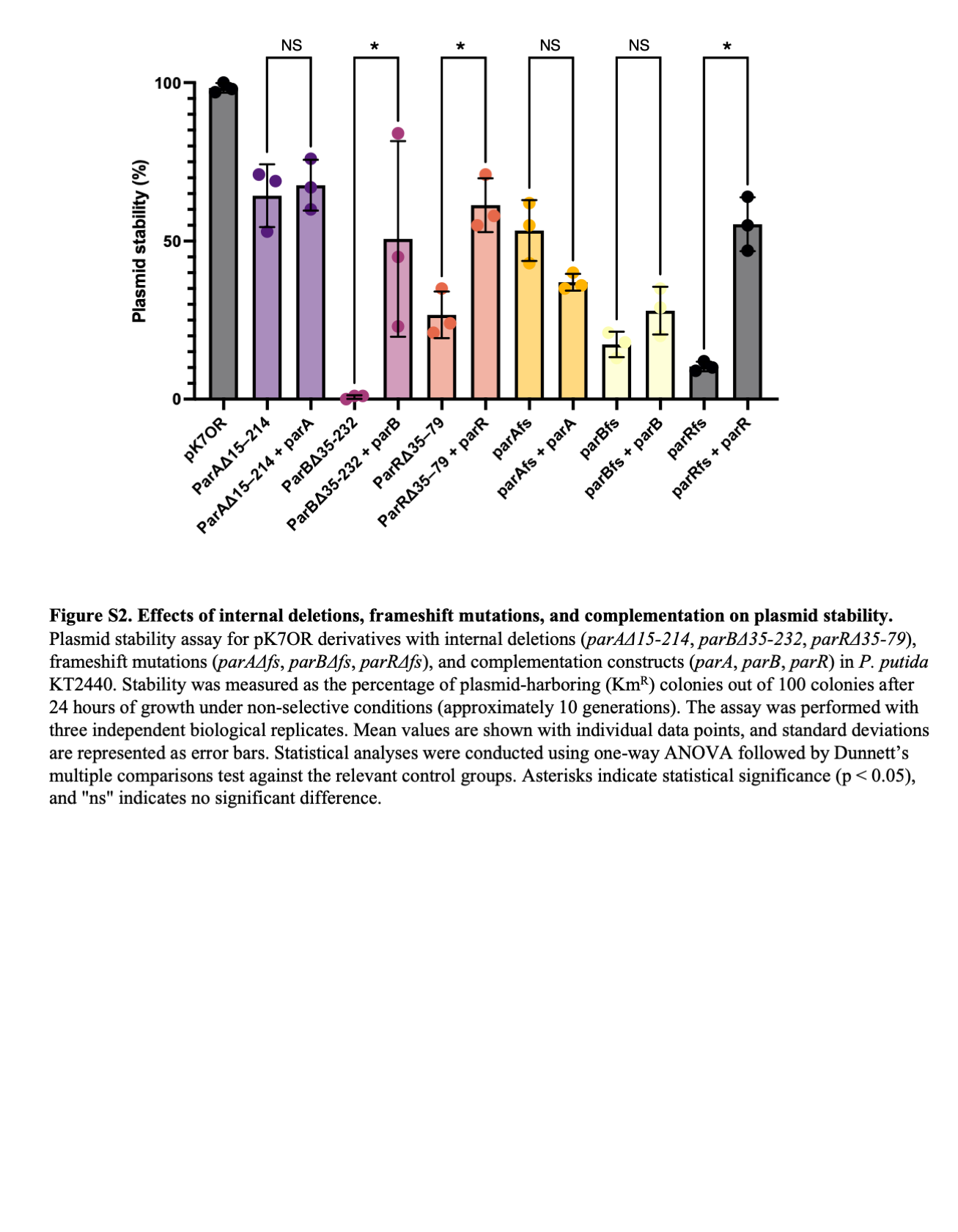

Supplement: Fig. S2 — Effects of internal deletions, frameshift mutations, and complementation on plasmid stability. [file mbio.01600-25-s0002.tiff]

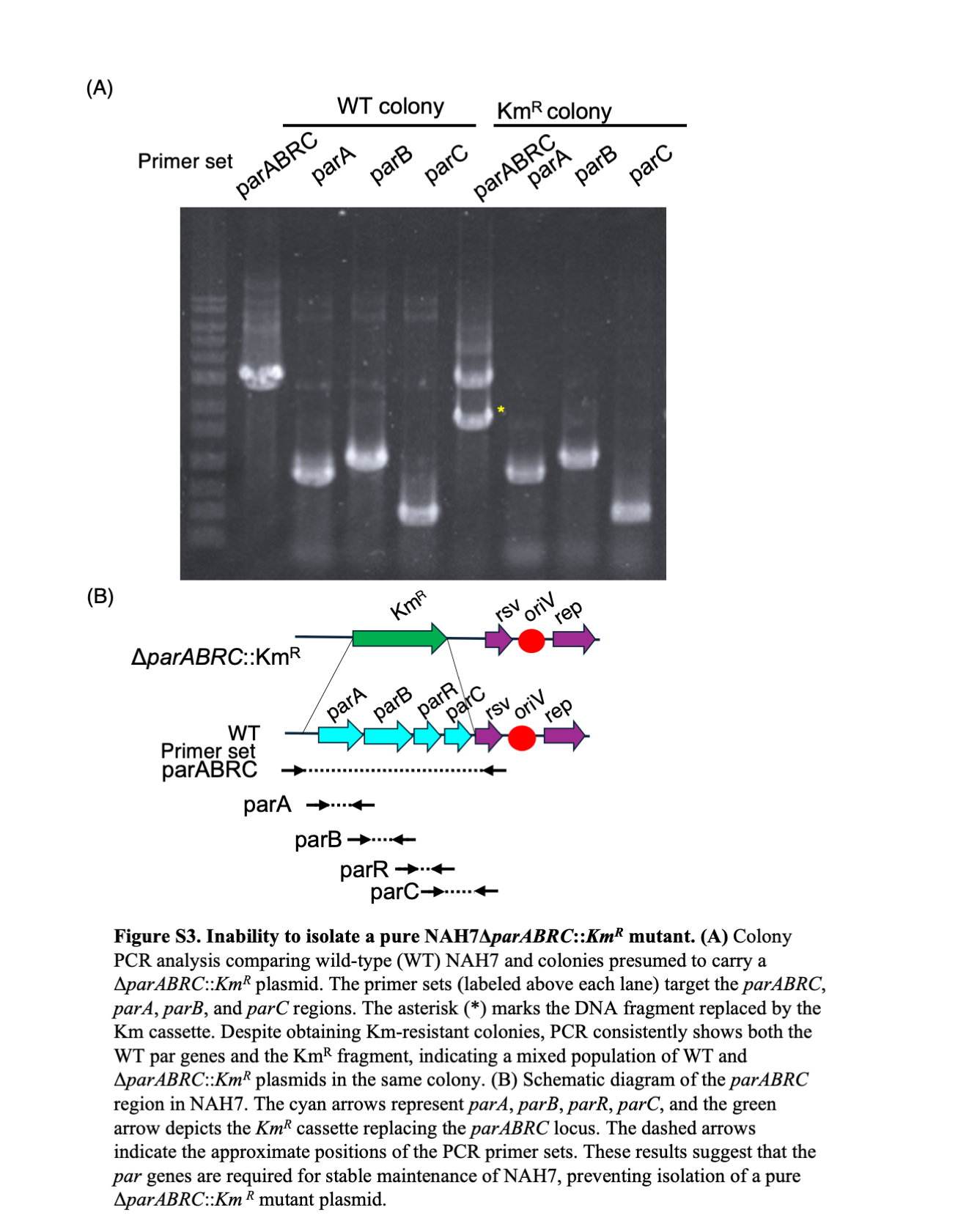

Supplement: Fig. S3 — Inability to isolate a pure NAH7ΔparABRC::KmR mutant. [file mbio.01600-25-s0003.tiff]

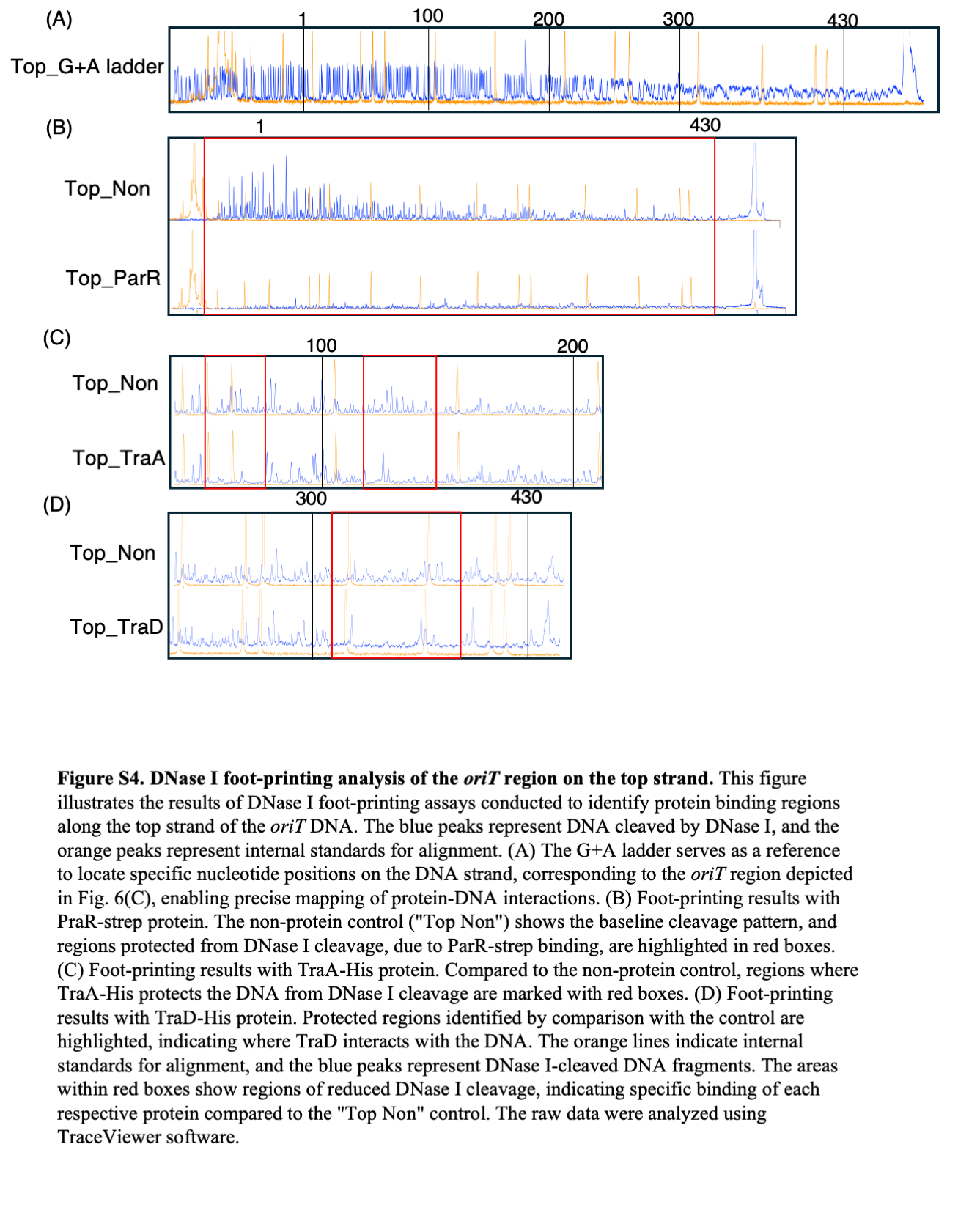

Supplement: Fig. S4 — DNase I footprinting analysis of the oriT region on the top strand. [file mbio.01600-25-s0004.tiff]

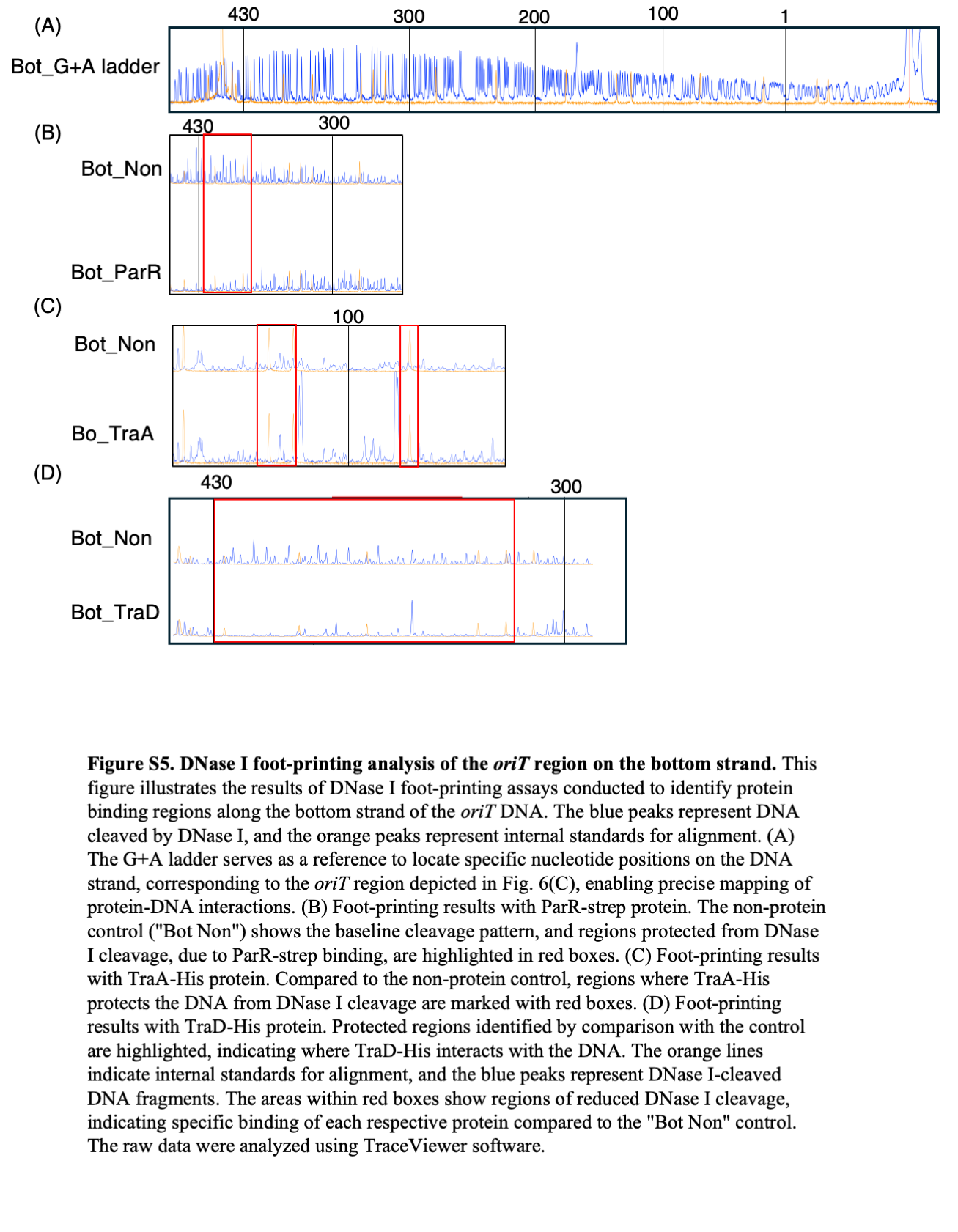

Supplement: Fig. S5 — DNase I footprinting analysis of the oriT region on the bottom strand. [file mbio.01600-25-s0005.tiff]

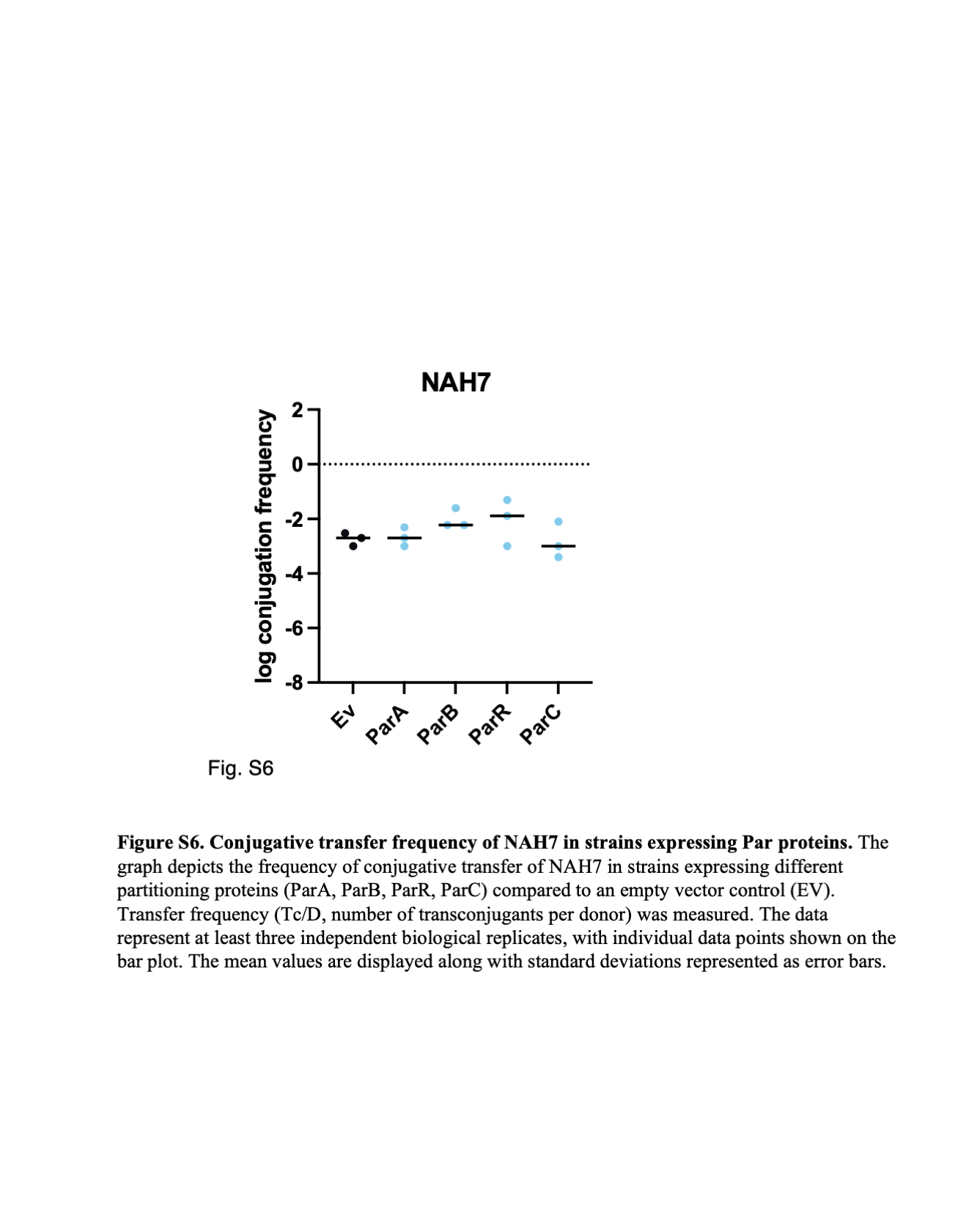

Supplement: Fig. S6 — Conjugative transfer frequency of NAH7 in strains expressing Par proteins. [file mbio.01600-25-s0006.tiff]

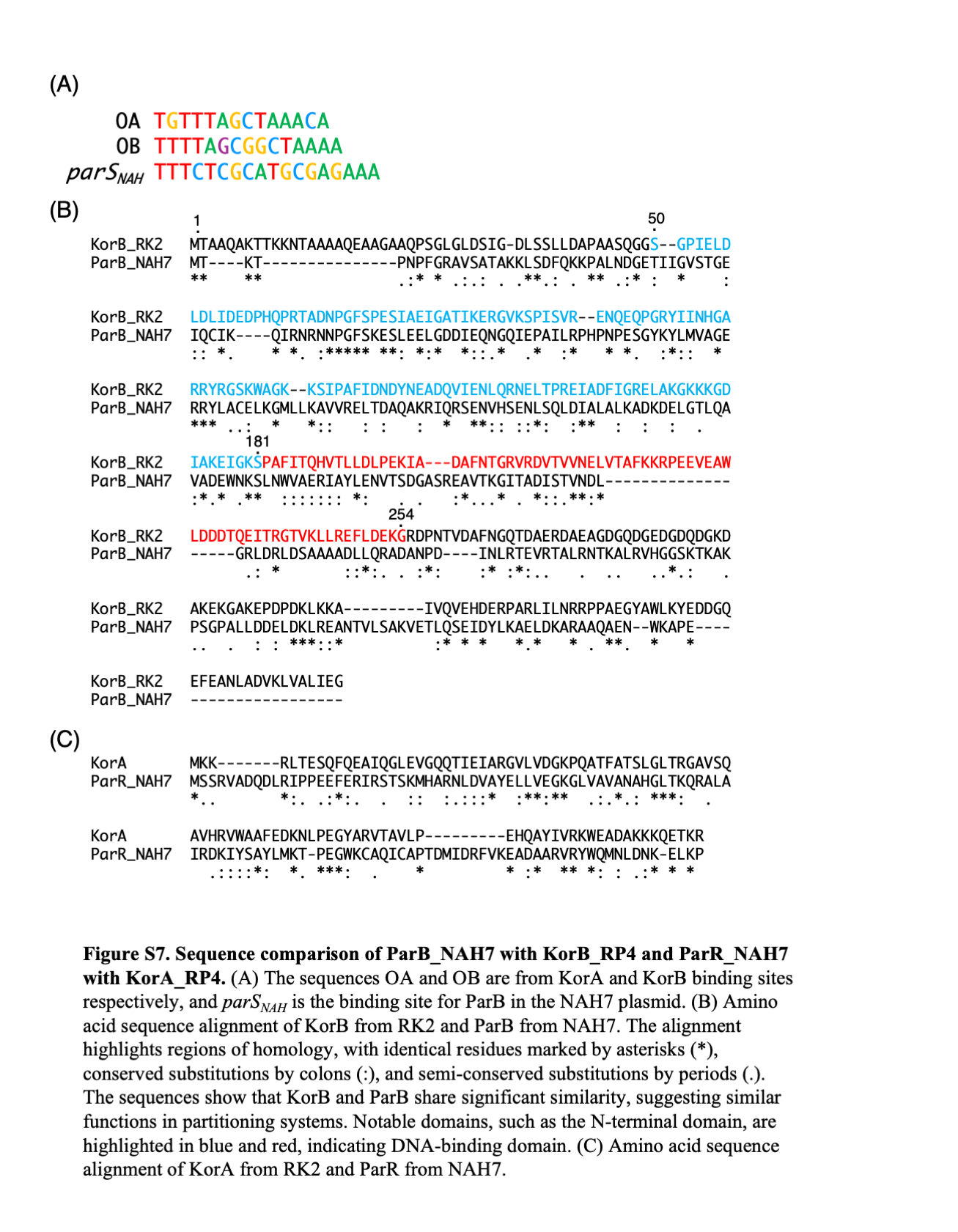

Supplement: Fig. S7 — Sequence comparison of ParB_NAH7 with KorB_RP4 and ParR_NAH7 with KorA_RP4. [file mbio.01600-25-s0007.tiff]

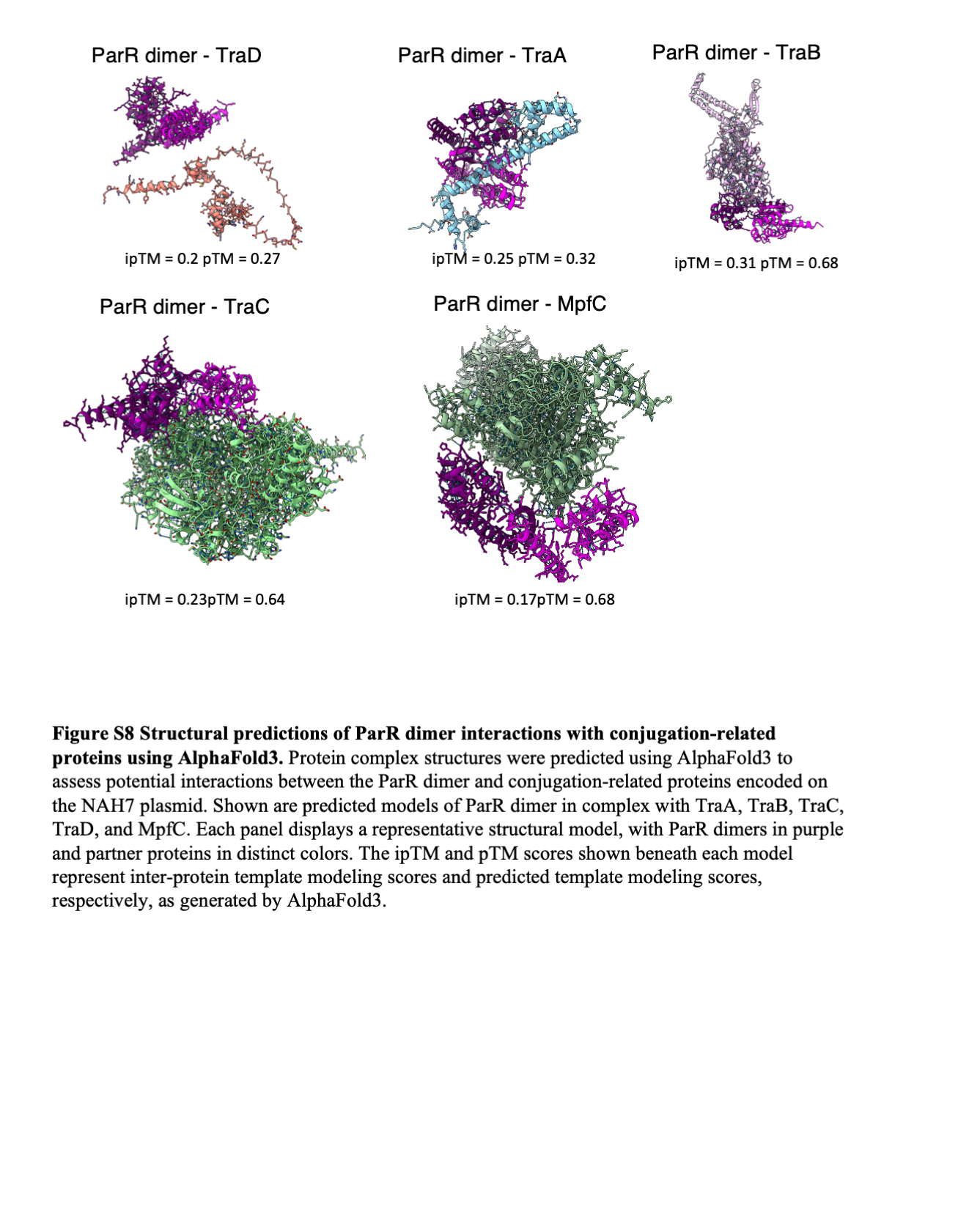

Supplement: Fig. S8 — Structural predictions of ParR dimer interactions with conjugation-related proteins using AlphaFold3. [file mbio.01600-25-s0008.tiff]

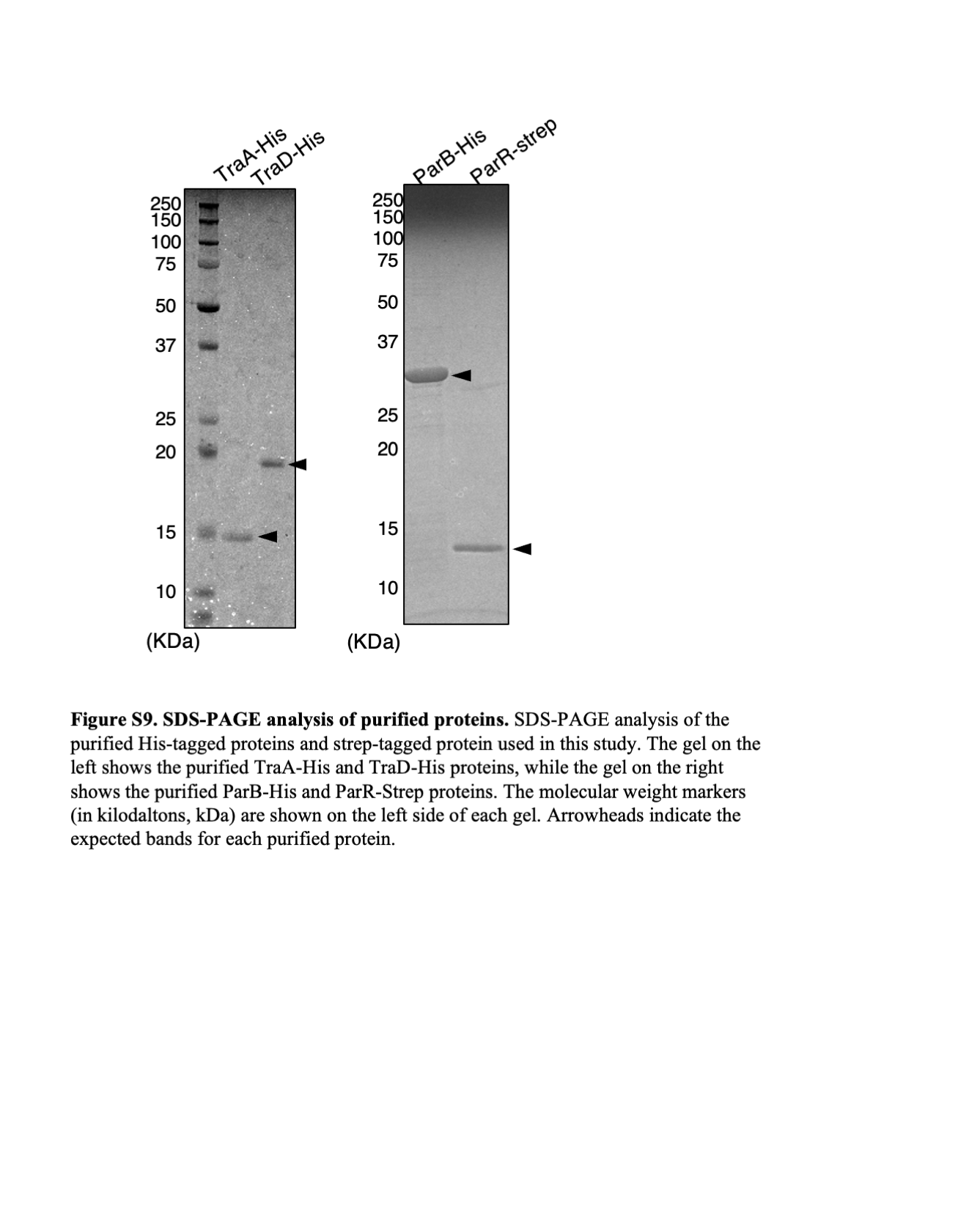

Supplement: Fig. S9 — SDS-PAGE analysis of purified proteins. [file mbio.01600-25-s0009.tiff]
